# A Review of Recent Studies on the Antioxidant and Anti-Infectious Properties of *Senna* Plants

**DOI:** 10.1155/2022/6025900

**Published:** 2022-02-04

**Authors:** Mohammed M. Alshehri, Cristina Quispe, Jesús Herrera-Bravo, Javad Sharifi-Rad, Sena Tutuncu, Elif Feyza Aydar, Cansu Topkaya, Zehra Mertdinc, Beraat Ozcelik, Mahima Aital, N. V. Anil Kumar, Natallia Lapava, Jovana Rajkovic, Andrea Ertani, Silvana Nicola, Prabhakar Semwal, Sakshi Painuli, Carlos González-Contreras, Miquel Martorell, Monica Butnariu, Iulia Cristina Bagiu, Radu Vasile Bagiu, Mrunal D. Barbhai, Manoj Kumar, Sevgi Durna Daştan, Daniela Calina, William C. Cho

**Affiliations:** ^1^Pharmaceutical Care Department, Ministry of National Guard-Health Affairs, Riyadh, Saudi Arabia; ^2^Facultad de Ciencias de la Salud, Universidad Arturo Prat, Avda. Arturo Prat 2120, Iquique 1110939, Chile; ^3^Departamento de Ciencias Básicas, Facultad de Ciencias, Universidad Santo Tomas, Chile; ^4^Center of Molecular Biology and Pharmacogenetics, Scientific and Technological Bioresource Nucleus, Universidad de La Frontera, Temuco 4811230, Chile; ^5^Facultad de Medicina, Universidad del Azuay, Cuenca, Ecuador; ^6^Department Food Engineering, Faculty of Chemical and Metallurgical Engineering, Istanbul Technical University, Maslak, 34469 Istanbul, Turkey; ^7^Bahçeşehir University-School of Applied Disciplines-Gastronomy and Culinary Arts, Beşiktaş, İstanbul 34022, Turkey; ^8^BIOACTIVE Research & Innovation Food Manufacturing Industry Trade Ltd. Co., Maslak, Istanbul 34469, Turkey; ^9^Department of Chemistry, Manipal Institute of Technology, Manipal Academy of Higher Education, Manipal 576104, India; ^10^Medicine Standardization Department of Vitebsk State Medical University, Belarus; ^11^Institute of Pharmacology, Clinical Pharmacology and Toxicology, Medical Faculty, University of Belgrade, 11129 Belgrade, Serbia; ^12^Department of Agricultural, Forest and Food Sciences, University of Turin, Italy; ^13^Department of Biotechnology, Graphic Era University, Dehradun, 248 001 Uttarakhand, India; ^14^Uttarakhand State Council for Science and Technology, 248 007, Dehradun, Uttarakhand, India; ^15^Himalayan Environmental Studies and Conservation Organization, 248 001, Dehradun, Uttarakhand, India; ^16^Department of Nutrition and Dietetics, Faculty of Pharmacy, University of Concepción, 4070386 Concepción, Chile; ^17^Centre for Healthy Living, University of Concepción, Concepción 4070386, Chile; ^18^Banat's University of Agricultural Sciences and Veterinary Medicine “King Michael I of Romania” from Timisoara, Timisoara, Romania; ^19^Victor Babes University of Medicine and Pharmacy of Timisoara Department of Microbiology, Timisoara, Romania; ^20^Multidisciplinary Research Center on Antimicrobial Resistance, Timisoara, Romania; ^21^Preventive Medicine Study Center, Timisoara, Romania; ^22^Chemical and Biochemical Processing Division, Central Institute for Research (ICAR) on Cotton Technology, 400019, Mumbai, India; ^23^Department of Biology, Faculty of Science, Sivas Cumhuriyet University, 58140 Sivas, Turkey; ^24^Beekeeping Development Application and Research Center, Sivas Cumhuriyet University, 58140 Sivas, Turkey; ^25^Department of Clinical Pharmacy, University of Medicine and Pharmacy of Craiova, 200349 Craiova, Romania; ^26^Department of Clinical Oncology, Queen Elizabeth Hospital, Kowloon, Hong Kong

## Abstract

The use of phytochemicals is gaining interest for the treatment of metabolic syndromes over the synthetic formulation of drugs. *Senna* is evolving as one of the important plants which have been vastly studied for its beneficial effects. Various parts of *Senna* species including the root, stem, leaves, and flower are found rich in numerous phytochemicals. *In vitro*, *in vivo*, and clinical experiments established that extracts from *Senna* plants have diverse beneficial effects by acting as a strong antioxidant and antimicrobial agent. In this review, *Senna* genus is comprehensively discussed in terms of its botanical characteristics, traditional use, geographic presence, and phytochemical profile. The bioactive compound richness contributes to the biological activity of *Senna* plant extracts. The review emphasizes on the *in vivo* and *in vitro* antioxidant and anti-infectious properties of the *Senna* plant. Preclinical studies confirmed the beneficial effects of the *Senna* plant extracts and its bioactive components in regard to the health-promoting activities. The safety, side effects, and therapeutic limitations of the *Senna* plant are also discussed in this review. Additional research is necessary to utilize the phenolic compounds towards its use as an alternative to pharmacological treatments and even as an ingredient in functional foods.

## 1. Introduction

*Senna*—a genus belonging to family *Fabaceae*, subfamily *Caesalpinioideae*, tribe *Cassieae* ser. *Aphyllae*—has roughly 350 species of tree shrubs and subshrubs [1, 2]. It was set apart from *Cassia* s. l. with the identification of three definite genera, viz., *Senna*, *Cassia* L. (s.s), and *Chamaecrista* Moench [3, 4]. This genus can be found in wide-ranging habitats, in distinct climatic conditions, latitudes, and continents such as America, Africa, and Oceania and to a minor extent in Asia and Pacific islands [5]. *Senna* plants colonized forests (both humid and dry), deserts (both cold and dry), and rock outcrops [6]. Some ornamental species are widely used for landscape gardening due to the attractive yellow inflorescences and the high adaptability in terms of soil and environmental conditions [7]. Recently, some species from desert climates were proposed to prevent or block desertification in arid zones. The use of *Cassia* species is reported in the ancient Ayurvedic literature as a laxative, antimalarial, relaxant, and anti-inflammatory [8]. To date, the genus is also commonly recognized for its biologically active compounds and medicinal properties [9, 10].

The cosmopolitan presence of the *Senna* genus and its medicinal properties lead to its various traditional medicinal uses and health-promoting effects. These beneficial effects of the *Senna* genus are contributed by the diverse group of phytoconstituents present in its leaves, stem, and seeds. By phytochemical research, more than 350 compounds were extracted from *Senna*, together with forty secondary metabolites extracted from *Senna spectabilis* (DC.) H.S.Irwin & Barneby. These phytochemicals majorly included classes of pentacyclic triterpenes and piperidine alkaloids displaying health-promoting properties [11]. Many of the parts such as leaves, pods, roots, and fruits of the natural plants have beneficial pharmacological properties against diseases. The studied pharmacological activities of *Senna* plants include anti-infectious, antioxidant, anticryptococcus, antitumor, antimutagenic, antiplasmodial, anti-inflammatory, anticancer, antidiabetic, wound healing, and antihelmintic activities [12, 13]. Some studies have shown the antidiabetic activity of *Senna* plants due to the content of phenols and flavonoids [14]. The antidiabetic effects have as mechanisms the decrease of the expression levels of different adipokines and the reduction of glucose absorption [15].

Its anti-infectious and antioxidant properties are established using various experiments, i.e., *in vitro* or *in vivo*.

The current review is focused on the traditional medicinal uses, phytoconstituents, antioxidant and anti-infectious properties, clinical trials, and toxicological data of S*enna* species.

## 2. Review Methodology

Information on the antioxidant and anti-infective pharmacological studies of *Senna* species has been collected from various scientific databases such as PubMed, ScienceDirect, and Google Scholar. The selected studies were analyzed for the phytochemical, antioxidant and anti-infective, toxicological aspects of *Senna* plants. The next MeSH keywords have been used for searching: “*Senna* Plant/growth & development,” “*Senna* Plant/metabolism,” “*Senna* Plant/chemistry,” “*Senna* Extract,” “Cassia/chemistry,” “Plant Extracts,” “Plant Extracts/chemistry,” “Oxidative Stress,” “Reactive Oxygen Species,” “Antioxidants,” “Antioxidants/chemistry,” “Malondialdehyde,” “Anti-Infective Agents/pharmacology “Antioxidants/pharmacology,” “Anti-Bacterial Agents,” “Anti-HIV Agents,” “Reverse Transcriptase Inhibitors,” “Antifungal “Agents/pharmacology,” “Antiprotozoal Agents/pharmacology,” “*Senna* Plant/toxicity,” “Animals,” and “Humans.” The scientific names of the *Senna* species were validated using the Plant List database and the chemical formulas with ChemSpider [16, 17].

## 3. Botanical Description and Distribution

Among the plants of the genus *Senna*, there is a semishrubby or shrubby habit, reaching 4-9 meters in height. *Senna* plants will tolerate moistly and very poorly draining soils in which it grows naturally. Giving a unique description of general botanical characteristics is tedious given the numerous species included in this genus. *Senna* has paripinnate compound leaves, with leaflets facing opposite, and globose, cylindrical, or clavate glands on rachis, petiole, or stalk [18]. The flowers are generally yellow and appear in dense racemes. It has large, lateral, terminal inflorescences with branched leafy panicles and can be up to 15–30 cm long. The flowers have fragrance and are made of 5 bristly bracts that usually are oval, 4–5 mm long, and caduceus and pedicles (2–3 mm). The sepals/calyx are unequal, oval to circular, coloured yellow-orange, and 5–7 mm long in size. The flower has 5 (uneven) golden-yellow-coloured petals and an ellipsoidal or spoon-like structure and is 2–3.5 cm in length. Anthers are opening by apical pores and a slit. It has sterile stamens that are 7 large and 3 small, while the pistil is curvy, slender, and hairless. The ovary is smooth and recurved with an inconspicuous style and stigma. The fruits of *Senna* are green in colour that turns black or dark with ripening, and their shape is cylindrical or column-like long pods. These pods are hard, end in a short, none splitting [7]. The size of the seeds is nearly 5 mm in diameter as they are brown coloured with flattened shapes.

The flowers of genus *Senna* present an interesting structural specialization that includes outstanding androecial diversity and several floral asymmetry patterns [7]. Classification of *Senna* flower traits becomes even more complicated due to its extraordinary level of specialization of the buzz-pollination. Ten stamens are present in heterantherous flowers of *Senna*, out of which 3 adaxial stamens are staminodial and the rest are fertile. These are further divided into two sets, viz., one set of four middle stamens from which the bees buzz and extract pollen, while another set includes 2-3 abaxial stamens, and the pollen from here is deposited on bees through the buzzing and is carried to the stigma of another flower [19]. *Senna* genus has 3-colporate pollen grains, ranging from size small to medium, and is euripalynic, radiosymmetric, and isopolar; however, the shape is oblates-spheroidic to prolate, nearly circular, and copli is long, subtriangular to triangular. Floral asymmetry is also due to the corolla and androecium. Extrafloral nectaries represent an “*archaic feature*” of numerous *Senna* species [5]. This appears in ca. 76% of the American species, several Australian species, scarcely in African, and none in Southeast Asian species. These glands secreting nectar can draw insects like ants that eat the nectar thus protecting the plant from the herbivores [20]. The fruits of *Senna* are long, enlarged, and tubular/cylindric, with the pods having 25-32 cm size, and the colour is black that has brown seeds equipped with pleurogram [11].

*Senna* can be propagated by seeds that remain viable for several years [21]. Most of the species of *Senna* require the scarification of the seeds to favour germination. The plant has numerous lateral roots and a robust primary root that contribute to the colonization of different substrates. Among the several species of *Senna* the series Aphyllae (Benth.) H.S.Irwin & Barneby is a taxonomically complex group of xeromorphic shrubs and subshrubs of the caesalpinioid legume *Senna* Mill., from arid, semiarid, and xerophilous areas of southern South America. Among all the *Senna* species, these seven are morphologically distinct. Fully grown mature plants are without leaves, and stems are junciform, green, and photosynthetic, while roots are woody and deep. These xerophytic attributes assist their survival in harsh conditions [22].

The monophyletic nature of *Senna* was revealed by phylogenetic investigations making it occupy the place next to *Cassia sensu stricto* and *Chamaecrista* [6], and all of these together form the subtribe *Cassiinae* are morphologically identified based on traits of their androecium, floral architecture, corolla, bracteoles, and fruits [23]. To date, taxonomy is not simply based on floral and vegetative characters, but on several other information, such as anatomy, cytology, serological, and molecular biology, that is useful for determining relationships and affinities among the *Senna* genus. DNA sequencing of various chloroplast gene sections of *Senna* plants (matK, rpL16, rpS16) depicted that majority of them are polyphyletic [5]. The chromosome counts exist only for about 20% of *Senna* species, with a prevalence of 2n = 28. There are also records of 2n = 22, 24, and 26 [24, 25] and records of polyploidy, such as 2n = 42, 56, and 112 in *Senna rugosa* (G.Don) H.S.Irwin & Barneby [26]; 2n = 56 in *Senna aversiflora* (Herbert) H.S.Irwin & Barneby; and 2n = 52 and 104 in *Senna gardneri* (Benth.) H.S.Irwin & Barneby [27]. Recently, Cordeiro and Felix [23] demonstrated that the karyotypic differences noted in *Senna*, either interspecific or intraspecific, are making this genus among the most representative taxa of the *Fabaceae* in several world territories [22].

**Figure 1:**
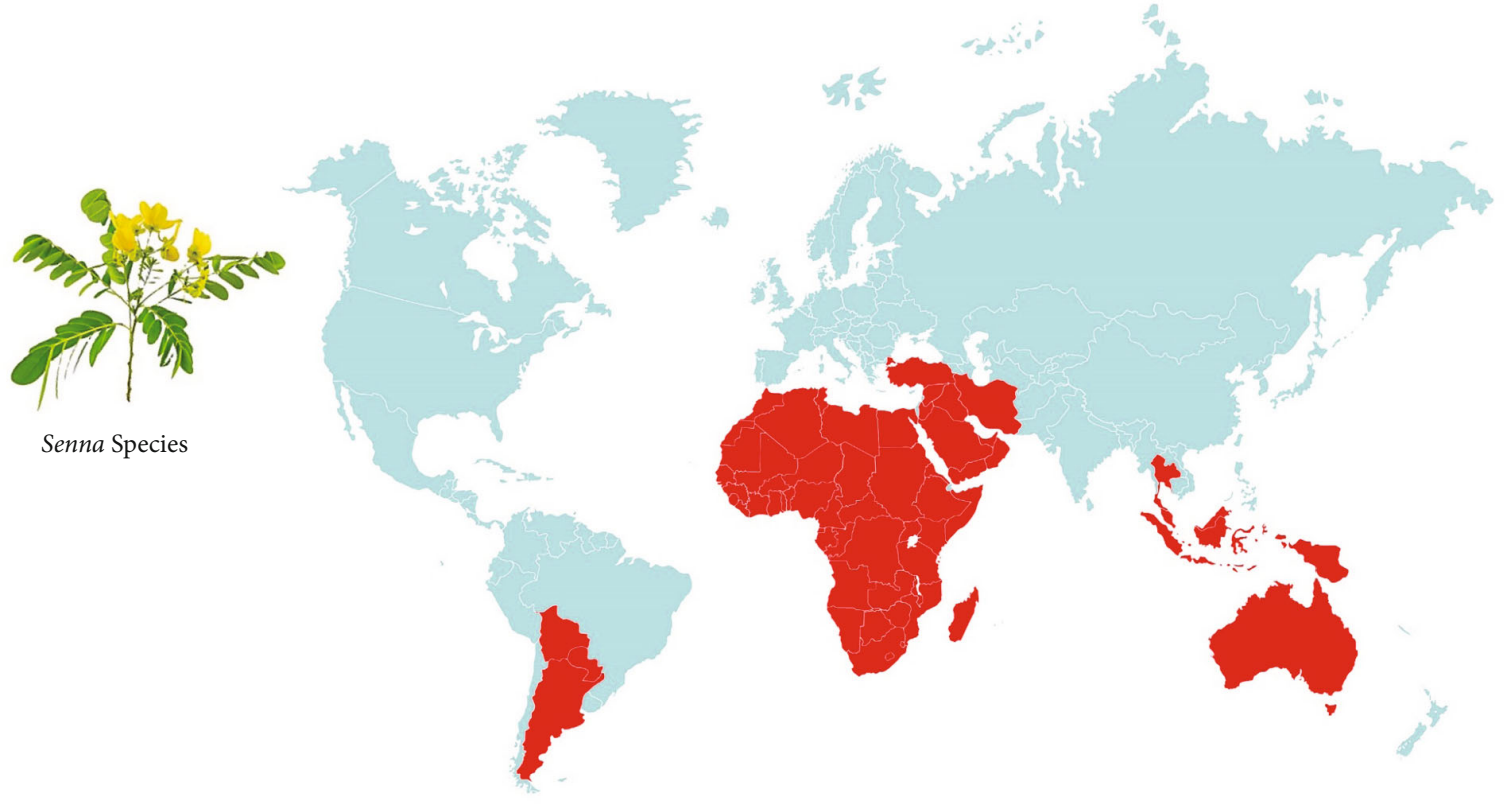
Geographical distribution of *Senna* species. All the regions where *Senna* plants are most common are highlighted in red

Plants of *Senna* genus are present in all the tropical regions and grow well on wasteland, river banks, damp/moist uncultivated fields, or similar areas in the low-lying coastal region; they also grow at places with altitudes up to 1000-1400 meters [28] (Figure 1).

*Senna*'s evolutionary history is also linked to the arid lands that this genus currently populates, such as deserts and xerophilous regions of South America in southern Bolivia, southeastern Paraguay, and central and northwestern Argentina [22]. Several types of research conducted in plants of genus *Senna*, growing in diverse climatic conditions, revealed a variation in phenotype between individuals within species that could arise from phenotypic plasticity.

Geographical separation and/or morphological variation among individuals of *Senna* causes the formation of species and subspecies in a different habitat, thanks to the adaptive strategies. America has the majority of *Senna* species (74%), followed by Australia with 13 percent of species and Africa and/or Madagascar having 10 percent, while only a few species are obtained from Near East, South-East Asia, and on the Pacific Islands [29]. Soladoye et al. [30] reported about 19 species in the West African floristic region with the whole 19 species in Nigeria and at least 8 species in South-Western Nigeria, with a high variety in habits, ranging from trees (approaching 34 m in height) to prostrate annual herbs. There are about 18 species of *Senna* in southern Africa, of which the majority is naturalized, but only *Senna italica* subsp. *arachoides* (Burch.) Lock and *Senna petersiana* (Bolle) Lock are native [31].

In Thailand, Larsen [32] studied *Senna* and stated that there are three native species, namely, *Senna timoriensis* (DC.) H.S.Irwin & Barneby, *Senna siamea* (Lam.) H.S.Irwin & Barneby, and *Senna garrettiana* (Craib) H.S.Irwin & Barneby, and fourteen exotic species, namely, *Senna alata* (L.) Roxb. (syn. *Cassia alata* L.), *Senna singueana* (Delile) Lock (syn. *Cassia singueana* Delile), *Senna alexandrina* Mill. (syn. *Cassia angustifolia* M.Vahl), *Senna bicapsularis* (L.) Roxb., *Senna hirsuta* (L.) H.S.Irwin & Barneby, *Senna fruticosa* (Mill.) H.S.Irwin & Barneby, *Senna occidentalis* (L.) Link, *Senna pallida* (Vahl) H.S.Irwin & Barneby, *Senna surattensis* (Burm.f.) H.S.Irwin & Barneby, *Senna septemtrionalis* (Viv.) H.S.Irwin & Barneby, *Senna sophera* (L.) Roxb., *S. spectabilis*, *Senna sulfurea* (Collad.) H.S.Irwin & Barneby, and *Senna tora* (L.) Roxb (syn. *Cassia tora* L.) [33].

## 4. Ethnobotanical Uses

*Senna* genus is widely used in southern countries in different spheres of life such as building, decoration, rituals, nutrition, poisons, and medicine. Some plants of *Senna* genus are used as building wood and as a shade plant and landscape ornamental [33, 34]. *S. alata* bark decoction has been applied by the west and east Africans while tribal mark incision and tattoo was making on to the cuts [12].

In Uganda *Senna obtusifolia* (L.) H.S.Irwin & Barneby is used as a good luck charm before travelling [35]. Shoots and leaves of *S. garrettiana* and *S. siamea* are cooked in a dish called kaeng khi lek (a kind of curry) which is found in two forms—with and without coconut milk [33].

Other species consumed as boiled vegetables along with chili sauce include *S. timoriensis* for its tender leaves and flowers and *S. sophera* for its tender fruits and shoots [33]. The crude pounded bark of *S. alata* is used as fish poison [36]. And the most popular usage of *Senna* genus is as a traditional medicine used as a remedy for a vast range of diseases in various countries and cultures (Table 1).

## 5. Phytoconstituents

Ahmed and Shohael [68] reported the presence of anthraquinones named aloe-emodin, chrysophanol, emodin, and rhein from the *S. alata* leaves. Bradley Morris et al. [3] studied the variation in the concentration of sennosides A and B from pods and leaves of *S. alata*, *S. alexandrina*, *Senna covesii* (A.Gray) H.S.Irwin & Barneby, *Senna angulata* (Vogel) H.S.Irwin & Barneby, *S. hirsuta*, *S. occidentalis*, and *Senna uniflora* (Mill.) H.S.Irwin & Barneby [3]. Essien et al. [69] isolated oils from hydrodistillation of *S. alata*, *S. hirsuta*, and *S. occidentalis*. The following compounds are reported after analyzing samples using GC-MS (gas chromatography-mass spectrometry) analysis, viz., ar-turmerone, *β*-caryophyllene, (E)-phytol, and 6,10,14-trimethyl-2-pentadecanone. (E)-Phytol and pentadecanal were the main components of *S. hirsuta* while *S. occidentalis* had (E)-phytol, hexadecanoic acid, and 6,10,14-trimethyl-2-pentadecanone. Epifano et al. [70] isolated madagascin (3-isopentenyloxyemodin) and 3-geranyloxyemodine from dried fruits and leave samples of *S. alexandrina*.

Ahmed et al. [71] isolated the flavonoids quercimeritrin, scutellarein, and rutin from the leaves. Arrieta-Baez et al. [72] reported the isolation of alizarin and purpurin from *S. alexandrina*.

New compounds of pyridine alkaloids (12′-hydroxy-8′-multijuguinol, 12′-hydroxy-7′-multijuguinol, methyl multijuguinate, 7′-multijuguinol, and 8′-multijuguinol) were isolated using leaves of *Senna multijuga* (Rich.) H.S.Irwin & Barneby by Francisco et al. [73]. Similarly, Serrano et al. [74] in leaves identified compounds like isolated 7′-multijuguinone and 12′-hydroxy-7′-multijuguinone. Vargas Rechia et al. [75] extracted from seed (aqueous) extract compounds, viz., galactomannan and *O*-acetyl-glucuronoarabinoxylan. Abegaz et al. [76] separated anthraquinones, emodin, floribundone-1, torosanin-9′, 10′-quinone, anhydrophlegmacin, and 9-(physcion-7′-yl)-5,10-dihydroxy-2-methoxy-7-methyl-1,4-anthraquinone from *Senna multiglandulosa* (Jacq.) H.S.Irwin & Barneby.

Alemayehu and Abegaz [77] reported the presence of physcion, torosachrysone, floribundone-1, anhydrophlegmacin, and 9-(physcion-7′-yl)-5,10-dihydroxy-2-methyl-7-methoxy-1,4-anthraquinone (isosengulone) from the seeds of *S. multiglandulosa*.

Essien et al. [78] identified the following volatile oils from the fruits of *S. hirsuta* and *S. occidentalis* by GC-MS analysis. Compounds identified in *S. hirsuta* are as follows: *α*-pinene, germacrene, camphene, selinene, *β*-pinene, valencene, viridiflorene, 2-tridecanone, p-cymene, *α*-muurolene, limonene, 1,8-cineole, (Z,Z)-*α*-earnesene, *γ*-terpinene, *β*-bisabolene, trans-*γ*-cadinene, *δ*-cadinene, methyl chavicol, (E)-*α*-bisabolene, isothymol methyl ether, occidentalol, methyl thymol, caryophyllene oxide, bornyl acetate, cedrol, 1,10-di-epicubenol, *α*-copaene, 1-epi-cubenol, cyperene, *τ*-cadinol, *β*-caryophyllene, *α*-cadinol, 2,5-dimethoxy-pcymene, valerianol, *α*-humulene, cyperotundone, pentadecanal, benzyl benzoate, and *γ*-muurolene. Compounds identified in *S. occidentalis* are as follows: *α*-pinene, selinene, *β*-pinene, valencene, myrcene, *α*-selinene, *α*-phellandrene, viridiflorene, *δ*-3-carene, p-cymene, limonene, *β*-himachalene, *β*-bisabolene, terpinolene, 1,8-cineole, linalool, 7-epi-*α*-selinene, *α*-terpineol, *δ*-cadinene, methyl chavicol, caryophyllene oxide, bornyl acetate, myrtenyl acetate, humulene epoxide II, *α*-terpinyl acetate, *α*-copaene, 1-epi-cubenol, daucene, *γ*-eudesmol, cyperene, *τ*-cadinol, *β*-caryophyllene, valerianol, trans-*α*-bergamotene, (Z)-6,7-dihydrofarnesol, *α*-humulene, *α*-patchoulene, alloaromadendrene, *γ*-himachalene, and *γ*-muurolene.

Maia et al. [79] from methanolic extracts of *S. gardneri* and *Senna georgica* H.S.Irwin & Barneby separated compounds, viz., vanillic acid, 3,4-dihydroxybenzoic acid, syringic acid, dihydromyricetin, rutin glucoside, quercetin diglucoside, rutin pentoside, kaempferol rhamnodiglucoside, quercetin glucoarabinoside, kaempferol diglucoside, ellagic acid, rutin, oxyresveratrol, methoxy oxyresveratrol, quercetin glucoside, rubrofusarin tetraglucoside, quercitrin, kaempferol rhamnoglucoside, rubrofusarin triglucoside, rubrofusarin gentobioside, myricetin, quercetin, rubrofusarin glucoside, and emodin.

Monteiro et al. [80] reported the preliminary investigation on the qualitative phytochemicals present in *Senna cana* (Nees & Mart.) H.S.Irwin & Barn and *Senna pendula* (Willd.) H.S.Irwin & Barneby and reported the presence of saponins, anthraquinones, triterpenoids, steroids, flavonols, flavones, tannins, and xanthones.

Barba et al. [81] extracted different compounds from the leaves of *Senna corymbosa* (Lam.) H.S.Irwin & Barneby and roots of *Senna lindheimeriana* (Scheele) H.S.Irwin & Barneby. They were chrysophanol, methoxyhydroquinone, emodin, 5,7′-biphyscion (floribundone-l), physcion, p-hydroxybenzaldehyde, hydroquinone monomethyl ether, 3-hydroxy-4-methoxyphenol, *β*-sitosterol, stigmasterol, and linoleic acid in *S. corymbose*; while *S. lindheimeriana* had chrysophanol, xanthorin, chrysophanol 8-methyl ether, emodin, questin, physcion, 1-hydroxy-3-methyl-2,6,7,8-tetramethoxy-9,10-anthraquinone, 3,4,3′5′-tetrahydroxystilbene (piceatannol), 4,2′,4′-trihydroxychalcone (isoliquiritigenin), 2,4,5-trimethoxyphenol, betulinic acid, and stigmasterol.

Zavala-Sánchez et al. [82] analyzed the GC-MS result from the *Senna crotalarioides* (Kunth) H.S.Irwin & Barneby leaf (chloroform) extracts and reported the following compounds. 1-ocyacosanol, 1-triacontanol, palmitic acid, beta-sitosterol, neophytadiene, 1-hexacosanol, and stigmasterol.

Alemayehu et al. [83] from the pods of *Senna didymobotrya* (Fresen.) H.S.Irwin & Barneby isolated compounds, namely, knipholone, emodin, chrysophanol, 10-hydroxy-10-(physcion-7′-yl)-chrysophanol anthrone, physcion, and 5,10-dihydroxy-2-methyl-9-(physcion-7′-yl)-1,4-anthraquinone.

Ochieng et al. [84] reported that the root extracts (ethyl acetate) resulted in nataloemodin-8-methyl ether, obtusifolin, 1,6-di-*O*-methylemodin, chrysophanol, physcion, physcion-10,10′-bianthrone, chrysophanol-10,10′-bianthrone, and stigmasterol. Rao et al. [85] extracted compounds, namely, kaempferol 3-*O*-*α*-L-rhamnopyranosyl (1→2)-*α*-L-rhamnopyranoside, kaempferol 3-O-rutinoside, and rutin from the flowers of *S. hirsuta*.

Silva et al. [86] identified the following compounds from *S. gardneri*, *Senna macranthera* (Collad.) H.S.Irwin & Barneby, *Senna splendida* (Vogel) H.S.Irwin & Barneby, and *Senna trachypus* (Benth.) H.S.Irwin & Barneby through GC-MS. *S. gardneri* containing succinic acid, glyceric acid, *β*-caryophyllene, malic acid, pyroglutamic acid, 3-hydroxy-3-methylglutaric acid, 3,4-dihydroxy benzoic acid, citric acid, neophytadiene, gluconic acid, hexadecanoic acid, linolenic acid methyl ester, phytol, quercetin, *α*-linolenic acid, linoleic acid, stearic acid, *α*-tocopherol, eicosanoic acid, squalene, tetracosanoic acid, *β*-sitosterol, stigmasterol, 1-triacontanol. *S. macranthera* contains succinic acid, *β*-caryophyllene, malic acid, pyroglutamic acid, eicosanoic acid, hexadecanoic acid, docosanoic acid, *α*-linolenic acid, phytol, linoleic acid, stearic acid, chrysin, squalene, trans-catechin, *β*-tocopherol, *α*-tocopherol, quercetin, stigmasterol, *β*-sitosterol, *β*-amyrin, 1-triacontanol, and *α*-amyrin.

*S. splendida* contains succinic acid, glyceric acid, pentanedioic acid, pyroglutamic acid, 3-hydroxy-3-methylglutaric acid, stearic acid, galactonic acid, gluconic acid, hexadecanoic acid, linoleic acid, *α*-tocopherol, linolenic acid methyl ester, phytol, *α*-linolenic acid, docosanoic acid, squalene, tetracosanoic acid, stigmasterol, *β*-sitosterol, quercetin, *β*-amyrin, 1-triacontanol, *α*-amyrin. *S. trachypus* contains succinic acid, linoleic acid, hexadecanoic acid, neophytadiene, linolenic acid ethyl ester, *α*-linolenic acid, galactonic acid, gluconic acid, eicosanoic acid, phytol, stearic acid, stigmasterol, *β*-sitosterol, docosanoic acid, squalene, tetracosanoic acid, *α*-tocopherol, quercetin, *β*-amyrin, 1-triacontanol, and triacontanoic acid. Gololo et al. [87] identified the phytol (3,7,11,15-tetramethyl-2-hexadecen-1-ol); 1,2-benzenedicarboxylic acid, mono (2-ethylheptyl) ester; n-tetracontane; 13-docosenamide; squalene (2,6,10,14,18,22-hexamethyltetracosane),1-heptacosanol; *α*-tocopherol-*β*-D-mannoside; 1,2-epoxynonadecane; stigmasterol; *γ*-sitosterol and lupeol from hexane extract of *Senna italica* Mill. leaves through GC-MS analysis.

Khalaf et al. [88] used aerial parts and isolated physcion, emodin, 2-methoxy-emodin-6-*O*-D-glucopyranoside, quercetin 3-O-L-rhamnopyranosyl-(16)-D-glucopyranoside (rutin), 1-hydroxy-2-acetyl-3-methyl-6-hydroxy-8-methoxynaphthalene (tinnevellin), and 1,6,8-trihydroxy-3-methoxy-9,10-dioxo-9,10-dihydroanthracene. Similarly, Madkour et al. [89] identified n-hexadecanoic acid, (Z,Z,Z)9,12,15-octadecadienoic acid, vitamin E, from hexane extract and 3-methyl-4-oxopentanoic acid, (E)-stilbene, and 2,6-di-tert-butylphenol from methylene chloride extract by GC-MS analysis. Mokgotho et al. [90] extracted 3,4′,5-trihydroxystilbene (resveratrol) from aqueous extracts of the roots.

Alemayehu et al. [91] isolated 1,8,1′,8′-tetrahydroxy-6′-methoxy-3,3′-dimethyl-(10,10′-bianthracen)-9,9′-dione (or chrysophanol-physcion), 1,8,1′,8′-tetrahydroxy-7′methoxy-3,3′-dimethyl-(10,10′-bianthracen)-9,9′-dione (or chrysophanol- isophyscion-10,10′-bianthrone) and 1,8,1′,8′-tetrahydroxy-7,7′-dimethoxy-3,3′-dimethyl-(10,10′-bianthracen)-9,9′-dione (or isophyscion-10,10′-bianthrone) from the leaves and root bark of *Senna longiracemosa* (Vatke) Lock. Branco et al. [92] communicated the presence of rubrofusarin (5,6-dihydroxy-8-methoxy-2-methylbenzo[g]cromen-4-one, 1) in *S. macranthera*. Klika et al. [93] confirmed the (2R,3S,4S,2^″^R,3^″^S)-guibourtinidol-(4*α*→8)-catechin (procyanidin) in root isolates.

Pires et al. [94] isolated mannose and galactose from the endosperm of *S. macranthera* seeds. Messana et al. [95] isolated 10-demethylflavasperone-10-sulphate, 10-demethylflavasperone, 10-demethylflavasperone-10-*O*-*β*-D-apiofuranosyl-(1→6)-*O*-*β*-D-glucopyranoside, and cassiapyrone-10-sulphate (7-methyl-10-demethylflavasperone-10-suophate); quinquangulin-6-O-*β*-D-apiofuranosyl-(l→6)-*O*-*β*-D-glucopyranoside, rubrofusarin-6-O-*β*-D-glucopyranoside, quinquangulin-6-O-*β*-D-glucopyranoside and chrysophanol dimethyl ether, chrysophanol, physcion, cis-3,3′,5,5′-tetrahydroxy-4-methoxystilbene, trans-3,3′,5,5′-tetrahydroxy-4-methoxystilbene, and cassiaside B from the root methanolic extracts [96]. de Macedo et al. [97] reported the presence of bianthrone glycoside, namely, martianine 1 (10,10′-il-chrysophanol-10-oxi-10,10′-bi-glucosyl) from the stalks of *Senna martiana* (Benth.) H.S.Irwin & Barneby.

Graham et al. [98] isolated quinquangulin and rubrofusarin from the stem and fruit extract (methanolic) of *Senna obliqua* (G.Don) H.S.Irwin & Barneby.

Pang et al. [99] communicated extractions from seeds of *S. obtusifolia* and those included obtusifolin-2-*O*-*β*-D-(6′-*O*-*α*, *β*-unsaturated butyryl)-glucopyranoside (1) and epi-9-dehydroxyeurotinone-*β*-D-glucopyranoside. Saidu et al. [100] described the existence of cardenolides, flavonoids, saponins, alkaloids and anthraquinones in the leaves of *S. occidentalis*.

Javaid et al. [101] extracted 1,3-benzenedicarboxylic acid, bis(2-ethylhexyl) ester, 9,10-dimethyltricyclo [4.2.1.1(2,5)]decane-9,10-diol, 2(2-hydroxy-2-propyl)-5-methyl-cyclohexanol, 1,2-benzenedicarboxylic acid mono(2-ethylhexyl) ester, 7-hydroxy-3,7-dimethyl-octanal, and 5,6,6-trimethyl5-(3-oxobut-1-enyl)-1-oxaspiro[2.5]octan-4-one from the aerial parts. Kim et al. [102] isolated N-methylmorpholine from the seeds.

Kumar et al. [103] identified rutin, quercetin, kaempferol, catechin, ferulic acid, gallic acid, caffeic acid, and coumaric acid using LC-MS (liquid chromatography-mass spectrometry).

Li et al. [104] isolated cycloccidentalic acids A and B, cycloccidentalisides I-V, quercetin, luteolin, eriodictyol, robtein, chrysoeriol, 3-methylquercetin, 7,4′-dihydroxy-3′-methoxyflavone, 7,3′,4′-trihydroxyflavone, 3-methoxy-7,3′,4′-trihydroxyflavone, chrysoeriol 5-methyl ether, 2′,3,4′,4-tetrahydroxychalcone, ajugasterone C, 20-hydroxyecdysone 2-acetate, 20-hydroxyecdysone 3-acetate, calonysterone, and poststerone. S. F. Li and S. L. Li [105] isolated cycloccidentalic acid C and cycloccidentaliside VI.

Ogunwande et al. [106] identified the (E)-geranyl acetone, hexahydrofarnesylacetone, and (E)-phytol acetate through GC-MS. Qin et al. [107] extracted nor-sesquiterpene, 3-isopropyl-1,6-dimethoxy-5-methyl-naphthalen-7-ol, and 2,7-dihydroxy-4-isopropyl-6-methyl-naphthalene-1-carbaldehyde. Singh et al. [108] reported the isolation of emodin, rhamnetin 3-neohesperidoside, chrysophanol, physcion, cassiollin, quercetin, 5,7,2′,4′-tetrahydroxyfavanol, *β*-sitosterol, and chrysophanol.

Tshikalange et al. [109] extracted luteolin from the seeds of *S. petersiana*. Gamal-Eldeen et al. [110] isolated 7-acetonyl-5-hydroxy-2-methylchromone (petersinone 1), 7-(propan-2′-ol-1′-yl)-5-hydroxy-2-methylchromone (petersinone 2), 5-methyl-3-(propan-2′-on-1′-yl) benzoic acid (petersinone 3), 5-(methoxymethyl)-3-(propan-2′-ol-1′-yl) benzoic acid (petersinone 4), glyceryl-1-tetracosanoate, and sistosterol-3-*β*-D-glycoside from the leaves. Coetzee et al. [111] extracted cassiaflavan-(4*α*→8)-epicatechin, cassiaflavan-(4*α*→8)-epigallocatechin, cassiaflavan-(4*β*→8)-epicatechin, cassiaflavan-(4*β*→8)-epigallocatechin, cassiaflavan-(4*β*→8)-gallocatechin, ent-cassiaflavan-(4*β*→8)-epicatechin, and cassiaflavan-(4*α*→6)-epicatechin from the bark. Ajiboye et al. [112] isolated *β*-elemene, phytol, caryophyllene oxide chrysophanol, 3-oxo-methyl ester, *α*-humulene, *β*-caryophyllene, rhein, emodin, and *α*-copaene from the leaves of *Senna podocarpa* (Guill. & Perr.) Lock.

Malmir et al. [113] isolated rhein, emodin, chrysophanol, physcion, and sennosides A and B from the hydroethanol extracts of leaves and roots. Genta-Jouve et al. [114] isolated schoepfins A and D from *Senna quinquangulata*, while Ogura et al. [115] isolated quinquangulin.

Mena-Rejón et al. [116] isolated 8,9-dihydroxy-3-methoxy-2,2,6-trimethyl-(2H)-anthracen-1-one (racemochrysone) from *Senna racemosa* (Mill.) H.S.Irwin & Barneby bark extracts (hexane extract). Sansores-Peraza et al. [117] isolated cassine and inositol methyl ether from the leaves. Dos Santos et al. [118] extracted compounds from the wood of *Senna reticulata* (Willd.) H.S.Irwin & Barneby, and they include chrysophanol, emodin, physcion, aloe-emodin, 1,3,8-trihydroxyanthraquinone, 3-methoxy-1,6,8-trihydroxyanthraquinone, chrysophanol-10,10′-bianthrone, stigmasterol, *α* and *β*-amyrin, *β*-sitosterol, and kaempferol. Barbosa et al. [119] isolated chrysophanol, physcion, quinquangulin, and rubrofusarin from the roots of *S. rugosa*.

Alemayehu et al. [120] isolated chrysophanol, physcion, emodin, floribundone-1,5,7′-physcion-fallacinol, 5,7′-physcion-physcion-10′-C-*α*-arabinopyranoside from the stem bark of *S. septemtrionalis*. Similarly from the pods, Alemayehu et al. [121] isolated bianthraquinone, 5,7′-physcion-fallacinol (1,1′,8,8′,-tetrahydroxy-6,6′-dimethoxy-3-methyl-3′-hydroxymethylene-5,7′-bianthracene-9,9′,10,10′-tetraone) chrysophanol, physcion, torosachrysone, emodin, floribundone-1, and torosanin-9′,10′-quinone. Ingkaninan et al. [122] isolated luteolin, cassia chromone (5-acetonyl-7-hydroxy-2-methylchromone), 4-(trans)-acetyl, 3,6,8-trihydroxy-3-methyldihydronaphthalenone, 5-acetonyl-7-hydroxy-2-hydroxymethyl-chromone, and 4-(cis)-acetyl-3,6,8-trihydroxy-3-methyldihydronaphthalenone from the leaves of *S. siamea*.

The leaves are also reported to contain barakol [123], cassiarins A and B [124], and chrobisiamone A [125].

The floral parts of *Senna* plants species are reported to have cassiarins C-E, 10,11-dihydroanhydrobarakol [126], and cassibiphenols A and B [127]. The compounds such as 1,1′,3,8,8′-pentahydroxy-3′,6-dimethyl [2,2′-bianthracene]-9,9′,10,10′-tetrone, 7-chloro-1,1′,6,8,8′-pentahydroxy-3,3′-dimethyl [2,2′-bianthracene]-9,9′,10,10′-tetrone, emodin, cassiamin A, chrysophanol, friedelin, physcion, and cycloart-25-en-3*β*,24-diol were isolated from the root [128, 129].

The stems of *Senna* plant species are identified with physcion, chrysophanol, betulinic acid, lupeol, and emodin [130, 131]. In other studies, Lü et al. [132–134] reported the extraction of chrysophanol, 1-[(*β*-D-glucopyranosyl-(1→6)-*O*-*β*-D-glucopyranosyl)oxy]-8-hydroxyl-3-methy-9,10-anthraquinone, chrysophanol-1-O-beta-D-glucopyranoside [132], sucrose, *β*-sitosterol, n-octacosanol, 2-methyl-5-2′-hydroxypropyl)-7-hydroxy-chromone-2′-O-*β*-D-glucopyranoside, piceatannol [133], and 1,8,10-trihydroxyl-1-*O*-*β*-D-glucopyranosyl-3-methyl-10-C (S)-*β*-D-glucopyranosyl-anthrone-9 [134] from stem. Hu et al. [135] isolated siamchromones A-G, 7-hydroxy-2-methyl-5-(2-oxopropyl)-4H-chromen-4-one, *O*-methylalloptaeroxylin, perforatic acid, uncinoside A, peucenin-7-methyl ether, 8-methyleugenitol, urachromone A, 11-hydroxy-sec-*O*-glucosylhamaudol, sec-*O*-glucosylhamaudol, barakol, 4-cis-acety1-3,6,8-trihydroxy-3-methyldihydronaphthalenone, and 2-methyl-5-(2′-hydroxypropy1)-7-hydroxychromone-2′-O-D-glucopyranoside from the stem. In an independent work, Ledwani and Singh [136] reported the isolation of 1,8-dihydroxy-3-methyl anthraquinone and cassiamin from stem. Li et al. [137] isolate 6-hydroxy-7-methoxy-3-(4-methoxyphenyl)-2H-chromen-2-one, 7-hydroxy-6-methoxy-3-(4-methoxyphenyl)-2H-chromen-2-one, piceatanno1, 2,2′,3,3′-tetrahydroxyldiphenylethylene, candenatenin E, kaempferol, quercetin, and nonin A from the stems.

Thengyai et al. [138] isolated lupeol, *β*-amyrin, *α*-amyrin, betulin, betulinic acid, and scopoletin from the stem bark.

Baez et al. [139] isolated rutin, quercetin, 5,7-dimethoxyrutin, aglycon 5,7-dimethoxyquercetin, D-3-*O*-methyl-chiro-inositol, and piceatannol from roots of *Senna skinneri* (Benth.) H.S.Irwin & Barneby. Also, Baez et al. [140] isolated 5,7-di-O-methylrutin and 5,7-di-O-methylquercetin from *S. skinneri* and quercetin and rutin from *Senna wislizeni* (A.Gray) H.S.Irwin & Barneby. Alemayehu et al. [141] separated different compounds from the seeds of *S. sophera*, and these included presengulone [9-(6′methoxy-3′-methyl-3′,8′,9′-trihydroxy-1′-oxo-1′,2′,3′,4′-tetrahydro-anthracene-7′yl)-5,10-dihydroxy-2-methoxy-7-methyl-1,4-anthraquinone], physcion bianthrone, xanthorin, floribundone-1, isosengulone, sengulone, and anhydrophlegmacin-9,10-quinones A2 and B2. Kharat et al. [142] extracted hexahydroxydiphenic acid and kaempferol from methanolic extract of leaves.

Malhotra and Misra [143] isolated 1,3,6,8-tetrahydroxy 2-methyl 7-vinyl anthraquinone (sopheranin), 3-sitosterol, chrysophanol, physcion, and emodin from the roots and flowers. Mondal et al. [144] isolated 2-(3,4-dihydroxy-phenyl)-3,5-dihydroxy-7-methoxy-chromen-4-one.

Mushtaq et al. [145] isolated palmitic acid, palmitoleic acid, oleic acid, phytol, neophytadiene, and solasodine from *S. sophera* and *S. tora*. *S. spectabilis* is one of plant widely studied and reported. Selegato et al. [11] have reviewed the chemical aspects of *S. spectabilis*. Silva et al. [146] isolated caffeine, lupeol, *α*-amyrin, *β*-amyrin, cycloeucalenol, friedelin, ursolic, oleanolic, and betulinic acids, sitosterol, and stigmasterol and their respective glucosides from the leaves. Lim et al. [147] isolated (+)-spectaline and iso-6-spectaline from the leaves.

For this plant, flowers are recognized by (-)-cassine, (-)-cassine, (-)-spectaline, and iso-6-spectaline [148–150]. Sriphong et al. [151] isolated 3(R)-benzoyloxy-2(R)-methyl-6(R)-(11′-oxododecyl)-piperidine, 5-hydroxy-2-methyl-6-(11′-oxododecyl)-pyridine, 5-hydroxy-2-methyl-6-(11′-oxododecyl)-pyridine N-oxide, and (-)-cassine from the flowers. Viegas Junior et al. [152] isolated (-)-7-hydroxycassine), (-)-cassine, (-)-spectaline, (-)-3-*O*-acetylspectaline, (-)-7-hydroxyspectaline and (-)-iso-6-spectaline, *β*-sitosterol, luteolin, 3-methoxyluteolin, betulinic acid, and trans-cinnamic acid from the green fruits and flowers, whereas few other researchers reported piperidine alkaloid (-)-3-*O*-acetylspectaline, (-)-3-*O*-acetyl-spectalin, (-)-spectaline cassine, (–)-3-*O*-acetylcassine, iso-6-cassine, (–)-3-*O*-acetylspectaline, (-)-cassine, and (-)-spectaline [153–157].

Maia et al. [79] isolated quercetin diglucoside from the leaves, methoxy oxyresveratrol from the roots, quercetin-3-O-rhamnoside-4′-O-glucoside from the flowers (2.885 g/kg), while the bark of *S. splendida* had quercetin rhamnoside. Valencia et al. [158] isolated 5-(3-formyl-4-hydroxyfenoxy)-2-hydroxybenzaldehyde from stems and leaves of *Senna stipulacea* (Aiton) H.S.Irwin & Barneby.

El-Sawi and Sleem [159] isolated quercetin 3-*O*-glucoside 7-*O*-rahmnoside, quercetin, and rutin from the leaves of *S. surattensis*. Anu and Madhusudana [160] isolated kleinioxanthrone-1 and 2 from the aerial sections of *S. tora* [161] while roots had kleinioxanthrone-3 and 4. el-Halawany et al. [162] isolated torachrysone 8-*O*-[*β*-D-glucopyranosyl (1→3)-*O*-*β*-D-glucopyranosyl (1→6)-*O*-*β*-D-glucopyranoside], toralactone 9-*O*-[*β*-D-glucopyranosyl-(1→3)-*O*-*β*-D-glucopyranosyl-(1→6)-*O*-*β*-D-glucopyranoside], aurantio-obtusin 6-*O*-b-D-glucoside, torachrysone 8-*O*-b-D-gentiobioside, toralactone 9-*O*-b-D-gentiobioside, 6-hydroxymusizin 8-*O*-b-D-glucoside, torachrysone tetraglucoside, rubrofusarin triglucoside, and chrysophanol triglucoside from ethanolic extract of the seed. In another work, Fathalla et al. [163] identified chrysophanol, chrysarobin, 10-hydroxy-5-methoxy-2-methyl-1,4-anthracenedione, rubrofusarin, parietin, griseoxanthone-B, isotorachrysone, and cumbiasin B from the seeds through GC-MS. Lee et al. [164] isolated rubrofusarin-6-*O*-*β*-D-gentiobioside, cassiaside, and toralactone-9-O-*β*-D-gentiobioside from the seeds.

Hatano et al. [165] isolated rubrofusarin-6-*O*-*β*-gentiobioside, cassiaside, cassiaside C, chrysophanol-1-O-*β*-tetraglucoside, torosachrysone-8-*O*-*β*-gentiobioside, cassiaside C2, rubrofusarin triglucoside, torachrysone tetraglucoside, demethylflavasperone gentiobioside, nor-rubrofusarin gentiobioside, torachrysone gentiobioside, and torachrysone apioglucoside from the seeds. Lee et al. [166, 167] extracted emodin, 7-methoxy-obtusifolin, chrysoobtusin, obtusin, aurantio-obtusin, chrysophanol, obtusifolin, physcion, cassiaside, rubrofusarin-6-O-gentiobiosideol, obtusifolin-2-glucoside, cassitoroside, toralactone-9-*O*-gentiobioside, chryso-obtusin-2-*O*-glucoside, physcion-8-*O*-gentiobioside, glucoaurantio-obtusin, and alaternin 2-*O*-*β*-D-glucopyranoside from the seeds. In an independent study, Park and Kim [168] isolated chryso-obtusin-6-glucoside, norrubrofusarin-6-glucoside, and obtusifolin-2-glucoside, using seeds. Cherng et al. [169] extracted aloe-emodin, emodin, chrysophanol, and rhein. Hyun et al. [170] extracted emodin, alaternin, gluco-aurantioobtusin, gluco-obtusifolin, cassiaside, cassitoroside, chrysophanol triglucoside, toralactone gentiobioside, questin, and 2-hydroxyemodin 1-methylether from the methanol extract. Jimenez-Coello et al. [171] isolated (8-hydroxymethylen)-trieicosanyl acetate from the *Senna villosa* (Mill.) H.S.Irwin & Barneby. Guzmán et al. [172] isolated (8-hydroxymethylen)-trieicosanyl acetate from the leaf extract (chloroform extract).

**Figure 2:**
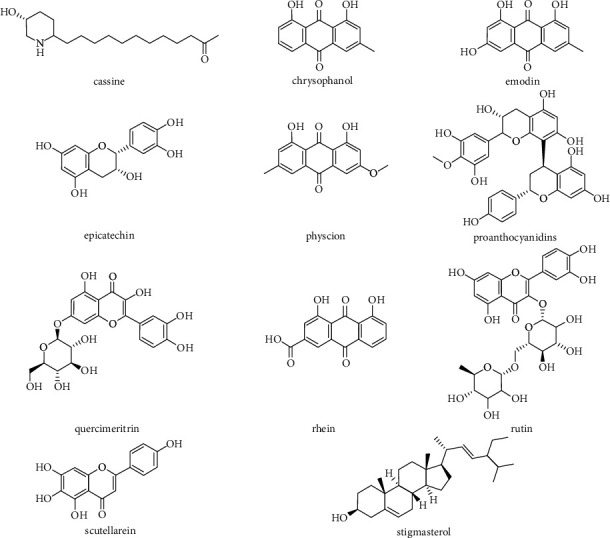
Chemical structures of mostly identified phytochemical compounds in *Senna* plants.

The chemical structures of some representative phytochemical compounds with therapeutic potencies in *Senna* plants are represented in Figure 2.

## 6. Antioxidant Activity of *Senna* Plants

Antioxidants are chemical compounds which are naturally present in food and also in human body [173–175]. These substances play a vital role for preventing cell damage caused by oxidative destruction as a result of free radical generation [176–178].

According to the literature, there are different pathways to acting as antioxidant agents [179, 180]:

1. Inhibiting the spread of free radicals or peroxide radicals by exchange of one or more protons
2. Reducing or blocking free radical formations with help of “metal chelating agents”
3. Reduction in reactive oxygen species (ROS) formation
4. Decreasing cellular ROS creation by hindering the oxidant enzymes
5. Influencing the complete antioxidant mechanism in the body by synergies of different antioxidant-rich ingredients

ROS are considered causative for various detrimental effects and persistent diseases like cancer, cardiovascular diseases (CVD), neurodegenerative dysfunction, like Alzheimer's, Parkinson's, and Huntington's diseases, sepsis, and diabetes [181–183].

The antioxidant activity of *Senna* genus was correlated with phenolic and flavonoid content which includes chemical compounds such as catechins, proanthocyanidins, scutellarein, rutin, quercimeritrin, kaempferol glycosides, rhein, chrysophanol, aloe-emodin, and physcion [184–186].

**Figure 3:**
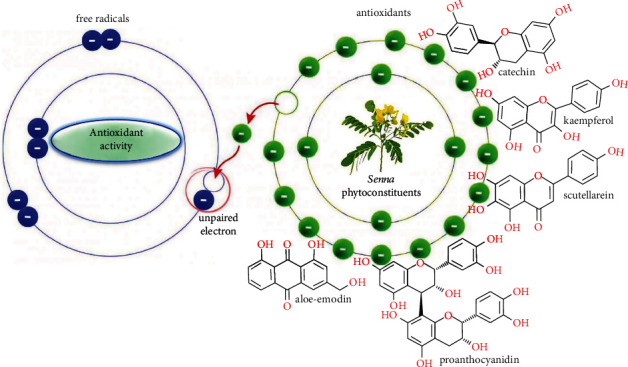
Antioxidant activity of bioactive compounds of *Senna* plants. The antioxidant bioactive molecules contained in *Senna* species neutralize free radicals by releasing electrons.

Neutralization of free radicals by the contained polyphenols justifies the antioxidant activities of the genus Senna. These polyphenols also quench singlet, and triplet oxygen, or decompose peroxides [187]. The antioxidant capacity and total polyphenol content of genus *Senna* were investigated by conducting both *in vitro* and *in vivo* experiments (Figure 3).

Commonly used *in vitro* techniques for determining the antioxidant activities of extracts are DPPH (2,2-diphenyl-1-picrylhydrazyl radical) and FRAP (ferric reducing antioxidant power) assay. The literature study indicates that various species under *Senna* genus were investigated using different methodologies, and they are indicated in Table 2. According to the study of Silva et al. [188] with four species of *Senna* from northeast Brazil, some of the phenolic compounds such as anthraquinones and flavonoids which are detected in the phytochemical screening especially in root extracts more than other parts can act as radical scavengers by donating hydrogen. They also mentioned that root extract of *S. trachypus* had a higher radical scavenging activity level than two standards (butylated hydroxyanisole (BHA) and quercetin) used in the assays.

Campos et al. [185] examined the chemical makeup of *Senna velutina* (Vogel) H.S.Irwin & Barneby leaf extracts (ethanol) and antioxidant activities with the DPPH method. In this study, IC_50_ (minimum sample concentration needed for scavenging 50 percent free radicals) values of the extract of *S. velutina* leaf extract; ascorbic acid and butylated hydroxytoluene (BHT) were found (6.3 *μ*g/mL, 2.6 *μ*g/mL, and 21.3 *μ*g/mL, respectively). This indicates that the antioxidant activity of *S. velutina* leaves is higher with a 3.5-fold than BHT but lower than ascorbic acid according to these results.

Ita and Ndukwe [189] studied the antioxidant activity of *S. alata* roots in different *in vitro* models. They used three different solvents such as acetone, ethanol, and water for extraction and measured its ferric reducing power, DPPH, ABTS (2,2′-azino-bis(3-ethylbenzothiazoline-6-sulfonic acid) radical-scavenging abilities, and metal chelating activity to determine antioxidant properties of roots. Researchers stated that ethanol extract had high amounts of total phenolics and flavonoids with values of 78.21 mg gallic acid equivalent (GAE)/g and 39.29 mg quercetin equivalent (QE)/g and exhibited the best antioxidant capacity in terms of DPPH and ABTS protocols. Besides, the aqueous extract showed more potential in metal chelating and reducing power. Khalaf et al. [88] analyzed the phenolic compounds, antioxidant, antimicrobial, and anticancer activities of *S. italica* aerial parts extracted using ethyl acetate and n-butanol. The researchers isolated and identified six compounds from this plant as they did bioguided fractionation. The names of these compounds are as follows: quercetin 3-*O*-*α*-L-rhamnopyranosyl-(1→6)-*β*-D-glucopyranoside (rutin), physcion, emodin, 1-hydroxy-2-acetyl-3-methyl-6-hydroxy-8-methoxynaphthalene (tinnevellin), 2-methoxy-emodin-6-*O*-*β*-D-glucopyranoside, and 1,6,8-trihydroxy-3-methoxy-9,10-dioxo-9,10-dihydroanthracene. Antioxidant activity was measured with ABTS method, and the ethyl acetate and n-butanol extracts showed 82.9% and 85.7% inhibition against ABTS radical, respectively, in comparison with ascorbic acid (89.2% inhibition). According to the literature, anthraquinone compounds which are already in this plant are given to their antioxidant potentials. Therefore, the researchers said that these anthraquinone-rich extracts (ethyl acetate and n-butanol) might be the reasons behind the high antiradical capacity. At last, it is noted that the aerial parts of *S. italica* may possess antioxidant activity and can serve as natural sources of antimicrobial and anticancer factors.

Phaiphan and Baharin [190] focused on determining effects of various extraction methods on some bioactive properties of *S. siamea* leaf. Researchers focused the study on comparing the solvent extraction with that of ultrasound-assisted extraction with regard to total phenolic content and antioxidant and antibacterial activity. In solvent extraction and ultrasound-assisted extraction (UAE), ethanol/water mixture (49%) and ethanol/water mixture (40%) were used, respectively, under the optimized conditions which were predetermined. The study showed that extracts from the ultrasound-assisted extraction had higher yield, total phenolic content (TPC), and antioxidant activities than those acquired from the solvent extraction. Furthermore, UAE extracts had greater antibacterial activity compared to solvent extracts. This can be attributed to the fact that the cavitational effect caused by ultrasound resulted in a more porous cell wall causing more release of phenolic bioactive in the solvent. It is evident from the literature that higher concentrations of bioactive have a direct correlation with antioxidant activity and antimicrobial activity. Similarly, Laghari et al. [191] investigated the comparison between 5 different extraction methods (microwave, Soxhlet, marination, reflux, and sonication) during the extraction of flavonoids to evaluate the antioxidative properties of *S. alexandrina.* As a result of this study, a greater quantity of flavonoids was obtained with microwave extraction in the aqueous ethanol (70%) fractions of *S. alexandrina* flowers and leaves.

In some of the studies, the antioxidant activity of some plants is compared with each other. In a study, five different medicinal plants (*S. alata*, *Eleusine indica* (L.) Gaertn., *Eremomastax speciosa* (Hochst.) Cufod., *Carica papaya* L., and *Polyscias fulva* (Hiern) Harms) collected from Cameroon were examined according to their scavenger activities against superoxide anion and hydrogen peroxide [192]. The results show that *S. alata* plant extracts at less than 12.5 *μ*g had the best scavenger activity with a 67% reduction in luminol-amplified chemiluminescence signal.

Navarro et al. [193] obtained and characterized (UPLC-DAD-EST-TQ-MS) phenolic extracts from *Petiveria alliaceae* L., *Phyllanthus niruri* L., and *S. reticulata*. Researchers also evaluated the antioxidant potential via conducting DPPH and ORAC (oxygen radical absorbance capacity) assay, and TPC was measured by the Folin-Ciocalteu method. Correlation analysis was carried out as well. It was reported that *P. niruri* has the highest phenolic content with 328.8 GAE/g, followed by *S. reticulata* with 79.3 GAE/g. In addition, *P. niruri* exhibited the best DPPH and ORAC values among these three plants. About the phenolic acid's characterization, for *S. reticulata*, the main compound was ferulic acid (52.6%) followed by 4-hydroxybenzoic acid, caffeic acid, vanillic acid, p-coumaric acid, and protocatechuic acid. *S. reticulata* had IC_50_ of 72.9 *μ*g/mL for DPPH and 2.68 mmol Trolox equivalents (TE)/g for ORAC. It was concluded that as TPC and UPLC increased ORAC values increased indicating a strong correlation.

Mak et al. [194] investigated the antioxidant capacity and antibacterial properties of ethanolic and distilled water extracts of hibiscus (*Hibiscus rosa-sinensis* L.) and *S. bicapsularis* flower. DPPH radical scavenging activity and FRAP were used as antioxidant assay while total phenolic content was analyzed by using the Folin-Ciocalteu method. DPPH inhibition values were 99.51 ± 0.2 for ethanol extracts and 96.51 ± 0.3 for aqueous extracts. The FRAP values found in the study were like 2403.15 ± 307.3 *μ*mol Fe (II)/100 g for ethanol extract and 1966.30 ± 12.7 for aqueous extract. Total phenolics were also determined in the study, and results are as follows: 26223.78 ± 450.3 mg GAE/100 g for ethanol extract and 9468.18 ± 91.9 mg GAE/100 g for aqueous extract. Researchers stated that these results were significantly different from each other and the other hibiscus flower extracts. Similarly, too many studies in literature, *Cassia* flower extracts (ethanolic) exhibited the highest TPC, total flavonoid, and flavonol content, which in turn had the highest DPPH radical scavenging activity. In addition to that, they suggested that all hibiscus and cassia flowers—because of their significant antioxidant activities—can be used as a natural preservative in formulations of new and creative functional products or nutraceuticals.

Channa et al. [195] studied medicinal properties, biochemical parameters, and antibacterial activity of *S. alata*'s various sections such as roots, stem, seed, leaves, and flower. To analyze the antioxidant capacity, the FRAP method was chosen and 80% methanol-water was used as a solvent. Researchers noted that the seeds were found rich in phenolic compounds compared to other parts. The seeds of *S. alata* contained a sufficient amount of total flavonoid whereas the leaves of the plant were quite rich in tannins. However, flowers were found the strongest antioxidative content. As a result, researchers suggested that these extracts have important potential for health benefits, so the plant needs to be isolated and test in detail.

Madubunyi and Ode [196] investigated the antioxidant potential of the *S. singueana* leaves with an *in vivo* malondialdehyde test. Malondialdehyde is an oxidative stress marker which is the end product of lipid peroxidation in the cells. In this study, all doses (0.25, 0.50, and 1.00 g/kg feed) of *S. singueana* extract significantly decreased malondialdehyde (MDA) level in the blood samples of test rats in comparison to the control group up to day 56. Similar to that study, treating rats using the methanolic extract of *S. singueana* root extracts was able to decrease malondialdehyde levels, the same as aspartate aminotransferase, alanine aminotransferase, and bilirubin level which are the indices of liver damage and lipid peroxidation, in all tissues especially in the liver and kidney [197].

## 7. Anti-infectious Activity of *Senna* Plants

### 7.1. Antibacterial and Antifungal

The most studied genus *Senna* for its anti-infectious activity was found to be *S. alata.* Different parts of *S. alata* are used as a vermicide, astringent, purgative, and expectorant and for treating skin diseases such as eczema, pruritus, itching, ulcers, scabies, and especially ringworm [198, 199]. Other species having antimicrobial activity are *S. spectabilis*, *S. alexandrina*, *S. occidentalis*, *S. podocarpa*, *S. tora*, *S. racemosa*, and *S. siamea.* The bioactive substances that provide bioactivity to genus *Senna* are steroids, flavonoids, anthraquinones, anthrones, and miscellaneous other compounds. They are located in the leaves, stems, roots, flowers, bark, seeds, and fruits.

Especially antibacterial and antifungal activities of *Senna* extracts are obtained from the extraction of leaves mostly. In the studies generally, minimum inhibitory concentration (MIC) is calculated which is described as the smallest concentration of sample necessary to prevent microbial growth. The MIC value of 100–200 *μ*g/mL is generally acceptable for plant materials [200]. Although the extracts of the parts of the genus *Senna* could not reach such MIC values, when the bioactive compounds are isolated from the extracts, MIC values decrease, and the antimicrobial properties increase [201]. Some of these bioactive components include stigmasterol, beta-sitosterol, kaempferol, luteolin, santal, alatonal, aloe-emodin, alquinone, chrysophanol, emodin, physcion, rhein, alarone, benzoquinone, coumarin, ellagitannin, naphthalene, phenolic acid, purine, xanthone, and cassine [202]. Anti-infectious effects of genus *Senna* are presented in Table 3.

The antifungal and antibacterial activity of the genus *Senna* varies depending on the species of the plant, the species of the microorganism, and the factors that affect the yield of the extraction process, such as the extraction method, the solvent used, the portion of the plant, and the secondary metabolite.

Ogunjobi and Abiala [203] investigated *in vitro* antimicrobial effect of different solvent extracts of *S. alata* leaves by using the agar well diffusion method. Except for *Aspergillus niger* inhibition, ethanol extract of *S. alata* showed a more inhibition zone than water extract. The best antimicrobial properties of *S. alata* ethanolic extract were shown in *A. niger* with a 25.2 mm zone of inhibition, and the least was *Salmonella typhimurium* with a 12.1 mm inhibition zone. *Escherichia coli* and *Candida albicans* had similar inhibition zone with 17.2 mm and 18.2 mm. In addition, ethanol extract of *S. alata* demonstrated effective antimicrobial activity of *Staphylococcus aureus* and *Aspergillus flavus* with 20.1 mm and 22.1 mm zone of inhibition. *S. alata* water extract observed the best effective antimicrobial characteristics against *A. niger* and *A. flavus* with 27.2 mm and 20.1. The other zone of inhibition was followed by *S. aureus* with 18.2 mm and *C. albicans* with 14.1 mm. The least effective antimicrobial activity was of aqueous extracts of *S. alata* against *E. coli* and *S. typhimurium* having inhibition zones of 10.2 mm and 10.1 mm, respectively.

Makinde et al. [204] research was about the methanol-water extract of *S. alata* leaves, and extract was assessed for antimicrobial activity by using a disc diffusion method (*in vitro* assay). The results indicated that *S. alata* leaves are more effective against fungi. *S. alata* phenolics and terpenoids, alkaloid salt, alkaloid base, and aqueous extract showed antimicrobial activity against *Microsporum canis*, *Blastomyces dermatitidis*, *Trichophyton mentagrophytes*, *C. albicans*, and *A. flavus* with 10–30 mm zone of inhibition. Phenolics and terpenoids, alkaloid salt, and alkaloid base extract of *S. alata* leaves had provided 5 mm of inhibition of *S. aureus*, *Corynebacterium parvum*, *Nocardia asteroides*, and *Clostridium septicum*; however, the aqueous extract had not shown antimicrobial activity of these bacteria. Phenolic and terpenoids and aqueous extract of *S. alata* leaf had 5–10 mm inhibition zone of *Dermatophilus congolensis*. Alkaloid salt and alkaloid base *S. alata* extract's inhibition zone of *D. congolensis* was 10–20 mm and 20–30 mm. Besides, *S. alata* antimicrobial activity was not observed against *Proteus vulgaris* and *Bacillus pumilus.*

Ehiowemwenguan et al. [205] examined the *S. alata* leaves and roots antimicrobial effect by using the cup plate agar diffusion method. Except for *Streptococcus pyogenes*, all inhibition zone is less than 5 mm; moreover, hot water extract, methanol extract, and acetone extract *S. alata* root and leaves did not differentiate among their inhibition zone. *S. pyogenes* had the highest zone inhibition at 5–6 mm both root and leaf extract independent of solvent type. *S. alata* root and leaves exhibited antimicrobial and antifungal reaction against *E. coli*, *Proteus mirabilis*, *Pseudomonas aeruginosa*, *Salmonella typhi*, *Shigella flexneri*, *S. aureus*, *A. flavus*, *A. niger*, *C. albicans*, and *Cryptococcus neoformans*. *S. alata* root extract's MIC level was changed (5–8 mg/mL) for bacteria species except for *S. pyogenes* (3 mg/mL); however, fungi needed more concentration, approximately 25-50 mg/mL for inhibition except for *C. neoformans* (6 mg/mL). The results of MIC level of leaves for bacteria were similar to root extract; yet, MIC range was between 6 and 10 mg/mL for bacteria. The leaf extract MIC was 35-50 mg/mL except *C. neoformans* (13 mg/mL). The minimum microbial concentration of *S. alata* leaf extract for bacteria was determined between 6 and 10 mg/mL, and fungi had more minimum microbial concentration at 25–50 mg/mL except *C. neoformans* (13 mg/mL). The minimum microbial concentration of root extract results was similar to leaf extract except for *S. pyogenes* (3 mg/mL) and *C. neoformans* (6 mg/mL).

Channa et al. [195] also detected antibacterial activity in root, stem, seeds, leaves, and flower extracts (methanol, ethanol, and water) of *S. alata.* In this study, a good diffusion method was used, and the results were between 8 and 34 mm. The least inhibition zone, 8 mm, was observed against *S. aureus* and *Klebsiella pneumoniae* by root-methanol, root-ethanol, leave-ethanol, stem-ethanol, and stem-water extraction. The maximum inhibition zone was observed against *E. coli* by leaves-methanol extraction. Furthermore, the results showed that flowers and leaves of *S. alata* possess antibacterial activity as compared to commercial drugs such as ciprofloxacin, penicillin, ampicillin, tetracycline, and gentamicin.

Sule et al. [206] experimented to determine *in vitro* antifungal activities of *S. alata* crude stem bark extract by using the agar diffusion method. Zones of inhibition were observed at 5 mg/mL and 10 mg/mL ethanol solvent of *S. alata* crude steam bark except for *T. mentagrophytes*. *T. mentagrophytes* has the highest inhibition zone with 17 mm at 5 mg/mL concentration. The inhibition zone followed the order as *Epidermophyton floccosum* with 15.5 mm, *Trichophyton verrucosum* with 15.0 mm, and *Microsporum canslaslomyces* with 12.0 mm at 5 mg/mL concentration. *T. verrucosum* and *E. floccosum* showed the best inhibition of zone with 21.0 mm and 20.5 mm at 10 mg/mL concentration. *M. canslaslomyces* had again the least zone of inhibition with 13.50 mm at a concentration of 10 mg/mL. However, a concentration of 10 mg/mL was effective against *T. mentagrophytes* with 19 mm inhibition of the zone. In addition, *T. mentagrophytes* was the only fungi that affected the inhibition at 2.5 mg/mL concentration with 10 mm zone. The MIC was evaluated at 5 mg/mL for all fungi. Minimum 5 mg/mL fungicidal concentration was appropriate for inhibition of fungi, except *E. floccosum*. The minimum fungicidal concentration of *E. floccosum* was determined at 10.0 mg/mL.

Abubacker et al. [198] conducted a study for *in vitro* antifungal properties of *S. alata* aqueous flower extracts, using three different fungal groups including fungi that produce aflatoxin (*A. flavus* and *Aspergillus parasiticus*), plant pathogenic fungi (*Fusarium oxysporum* and *Helminthosporium oryzae*), and human pathogenic fungi (*C. albicans* and *Microsporum audouinii*). The results highlighted the strong antifungal activity of *S. alata.* While 15 mg/mL of flower extract concentration provides 100% inhibition of all the fungus, 10 mg/mL was enough in inhibiting *A. flavus.* The MIC values of the flower extract of *S. alata* ranged from 5.75 to 8.0 mg/mL.

In a different investigation, Timothy et al. [207] assessed leaf extracts of *S. alata* (aqueous and ethanol) against five pathogenic fungi which are *C. albicans*, *M. canis*, *T. mentagrophyte*, *Penicillium notatum*, and *A. niger.* According to the calculated zones of inhibition, there was no inhibition for water extract of leaves whereas ethanol extracts exhibited inhibition for all tested microorganisms. Furthermore, MIC of ethanol extracts for all tested fungi was lower than the water extract indicating that ethanol extract includes more bioactive compounds than the water extract. The reason ethanol is being more effective than the water was told to be because of the presence of anthraquinone which is not found in the water extraction. Intense antifungal activities of *S. alata* were depicted from the study outcomes.

Wuthi-udomlert et al. [208] remarked on the importance of anthraquinone derivatives in the *in vitro* evaluation. Anthraquinone glycosides including emodin, rhein, and chrysophanol found in *S. alata* are the source of laxative effects. In the study, extraction of leaves is obtained in five different ways using anthraquinone aglycone, anthraquinone glycoside, anthraquinone aglycone from glycosidic fraction, crude ethanol, and anthraquinone aglycone from crude ethanol extract. Extraction yields were monitored by thin-layer chromatography, and the highest yield is obtained from crude ethanol extraction which was 34.94% *w*/*w*. As a result of the *in vitro* antifungal activity against *Trichophyton rubrum*, *T. mentagrophytes*, *E. floccosum*, and *Microsporum gypseum* by diffusion and broth dilution methods, anthraquinone aglycone from glycosidic fraction presented greater activity among five different extracts.

Phongpaichit et al.'s [209] experiment was about antifungal activities of *S. alata* and *S. tora*. Except *Penicillium marneffei*, 10 mg/mL methanolic extract obtained from leaves of *S. alata* and *S. tora* were enough in inhibiting all the *M. gypseum*, and *T. rubrum*. 10 mg/mL *S. alata* leaves inhibited only 77% of *P. marneffei*; still, *S. tora* extract was sufficient to inhibit all *P. marneffei.* In addition, IC_50_ result of *T. rubrum* followed the order as *S. alata* at 0.5 mg/mL and *S. tora* at 1.2 mg/mL. *S. alata* with 0.8 mg/mL IC_50_ value was the best inhibition for *M. gypseum*; also *S. tora* have an IC_50_ value at 1.8 mg/mL. The IC_50_ values of *P. marneffei* of *S. alata* and *S. tora* were 6.6 mg/mL and 1.8 mg/mL, respectively.

Malmir et al. [113] drew attention to the bioactive substance called “rhein” isolated from *S. podocarpa* root hydroethanol extract. In their study, *S. podocarpa* root extracts were evaluated for *in vitro* anti-*Neisseria gonorrhoeae* activity. Gonorrhoea is a widespread sexually transmitted infectious disease induced by *N. gonorrhoeae* bacterium infection. *N. gonorrhoeae* infects the mucous membranes of the reproductive organs that include the fallopian tubes, uterus, and cervix in women, while in men and boys it infects the urethra. *N. gonorrhea* can also harm the mucous membranes of the mouth, throat, and eyes [210]. *S. podocarpa* root demonstrated anti-*N. gonorrhoeae* activity against all strains. MIC ranged from 100 to 400 mg/L. The most active fractions having 50–100 mg/L MIC values, had rhein, emodin, chrysophanol, and physician as their key compounds as detected by LC-UV/DAD cochromatography with reference standards. Among all the isolates, rhein (MIC: 3.13 mg/L against all test strains) was the most effective. In addition to rhein, Sansores-Peraza et al. [117] highlighted the antibacterial and antifungal activity of cassine, isolated from *S. racemosa*, with MIC of 2.5 mg/mL against *S. aureus* and *Bacillus subtilis* and 5.0 mg/mL for *C. albicans.*

Albayrak et al. [211] indicated that infusion of *S. alexandrina* leaves is the only herb that has antibacterial effect against *Bacillus cereus* among infusions of eight plants in Turkey which are *Foeniculum vulgare* Mill. (fennel), *Pimpinella anisum* L. (anise), *Laurus nobilis* L. (laurel), *Tilia × europaea* L. (linden tea), *Urtica dioica* L. (nettle), *Petroselinum crispum* (Mill.) Fuss (parsley), and *Anethum graveolens* L. (dill). In the study, they extracted *S. alexandrina* leaves by four methods which are methanol extraction, infusion, decoction, and hydrosol. The *in vitro* antimicrobial activities of *S. alexandrina* leaves were evaluated, and the results showed that infusion of *Senna* leaves has antibacterial effect against *B. cereus* and methanol extracts of *Senna* have antibacterial activity against *B. cereus* and *P. aeruginosa.*

As a result of *in vitro* antibacterial analysis conducted by Jain et al. [212], although *Klebsiella aerogenes* exhibited resistance to all extracts, ethanol extracts of flowers and pods of *S. occidentalis* provide inhibition of growth of *E. coli* and *P. vulgaris*. In addition, a descending sort among bioactive compounds according to the antibacterial activities against test bacteria which are *E. coli*, *K. aerogenes*, *P. vulgaris*, and *S. aureus* was reported as anthraquinones>sennosides>flavonoids. Antifungal activity of ethanol extracts of *S. occidentalis* was found to be higher than the antibacterial activity. Among the metabolite-rich fractions, the maximum inhibition was shown by sennosides against *A. flavus*, followed by anthraquinones and flavonoids against *Curvularia lunata*.

### 7.2. Antiviral

Antiviral activity of genus *Senna* is generally found quite low; however, the extraction yield and the isolation of bioactive compounds provide an increase in the antiviral activity.

Jain et al. [212] investigated the antimicrobial, antitumor, and antiviral activity of ethanol extracts of *S. occidentalis.* They conducted *in vitro* analysis for antiviral and *in vivo* analysis for antitumor activity. The antiviral activity against *Herpes simplex* was quite inadequate; the reduction factor of titre was found 10 *μ*g/mL. In addition, *S. occidentalis* did not exhibit any antitumor activity or cytotoxicity.

Ogbole et al. [213] highlighted the antiviral agents that *S. siamea* includes which are lupenone, lupeol, betulinic acid, chrysophanol, physicon, and *β*-sitosterol glucoside. Among tested anthraquinones and triterpenoids, lupeol was the most effective constituent against poliovirus having 0.014 *μ*g/mL of IC_50_ value. Antipoliovirus, antitobacco mosaic virus, and anti-HIV-1 effects were observed in the extract of *S. siamea* stem bark [135, 214].

Another genus which is analyzed for the antiviral activity is *S. alata.* Shaheen et al. [215] determined the antiviral activity of methanol, chloroform, ethyl acetate, n-butanol, and aqueous extracts of *S. alata* by *in vitro* and *in vivo* experiments. The results justified the antiviral activity of *Senna*; all extracts exhibited antiviral effects against cardiac coxsackievirus B3. As a result of *in vitro* analysis, the therapeutic index varied between 0.2 and 12. *In vivo*, virus titer values were between 0 log_10_ and 2.5 log_10_. Both *in vitro* and *in vivo* analyses exhibited that the most effective extracts against cardiac coxsackievirus B3 were aqueous extracts. Woradulayapinij et al. [216] investigated *in vitro* HIV-1 reverse transcriptase inhibitory activity of ethanol and water extracts of aerial part of *S. alata*. Even though the results were quite close to each other, water extract depicted higher activity than the ethanol extract; inhibition ratios were 37 and 35.86% for water and ethanol extracts, respectively.

### 7.3. Antiprotozoal

Numerous studies reported antiprotozoal activities of genus *Senna.* de Castro et al. [201] conducted a study about the schistosomicidal activity of *S. spectabilis* flower extracts. *Schistosoma* is an intestinal parasite that causes a chronic disease called Schistosomiasis. The disease has been reported in 78 countries; especially, 90% of the cases have been reported in Africa where access to safe drinking water is a challenge. According to the WHO [217], at least 229 million people needed the treatment of *Schistosoma* in 2018. de Castro et al. [201] extracted and isolated (-)-cassine and (-)-spectaline substances from *S. spectabilis* flowers. *In vitro* activity of extracts, their fractions, and the mixture of (-)-cassine and (-)-spectaline against *S. mansoni* worms were analyzed. Obtained data indicated that the mixture of (-)-cassine and (-)-spectaline exhibited a multitarget mechanism against the excretory activity, tegument lesions, and neuromotor activity. It also showed a toxic effect on the larval period of cercariae. Therefore, *S. spectabilis* flower extracts (-)-cassine and (-)-spectaline have a great potential for their schistosomicidal activity. Furthermore, de Albuquerque Melo et al. [218] mentioned about leishmanicidal activity of *S. spectabilis* and the two major alkaloidal metabolites (−)-cassine/(−)-spectaline. Caamal-Fuentes et al. [219] studied antiprotozoal properties of *S. racemosa* against *Giardia intestinalis* and observed that methanolic extracts of *S. racemosa* bark in both *in vitro* and *in vivo* experiments had activity against *G. intestinalis* [219, 220]. Eguale et al. [221] mentioned the *in vitro* anthelmintic activity of *S. occidentalis*, and the extract concentration required to inhibit 50% (ED50) of the eggs of *Haemonchus contortus* was found to be 0.13 mg/mL and 0.17 mg/mL for aqueous and hydroalcoholic extracts, respectively.

**Figure 4:**
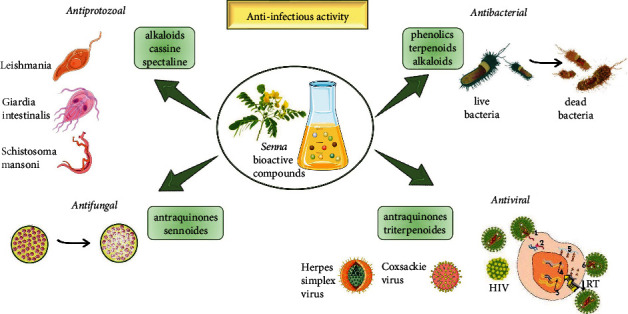
Anti-infectious properties of the most representative bioactive compounds of *Senna* plants. Botanical molecules such us alkaloids, sennoides, anthraquinones, phenolics, terpenoids, alkaloids, and triterpenoids have anti-infectious activity against bacteria, fungus, protozoa, and viruses (HIV, Coxsackie, and Herpes simplex).

A scheme with anti-infectious properties of *Senna* plants is summarized in Figure 4.

### 7.4. Other Biological Properties

Villaseñor et al. [222] also conducted research on *S. alata* leaf extracts with hexane, chloroform, and ethyl acetate to investigate antimutagenic, antifungal, analgesic, anti-inflammatory, and hypoglycemic activities. Chloroform extract exhibited a reduction in the mutagenic activity of tetracycline by 65.8% at a dosage of 2 mg/20 g mouse as a result of the *in vivo* analysis. Against fungi, *T. mentagrophytes* chloroform extract was the most effective. The hexane extract was having the highest analgesic property which provides a decrease of 59.9% at a dosage of 5 mg/20 g mouse among other extracts. The analgesic activity of hexane extract was similar to the activity of mefenamic acid which is a widely known analgesic. For the anti-inflammatory activity, all three extracts are observed hourly, for three hours. At the end of three hours, hexane and ethyl acetate extracts demonstrated 65.5% and 68.2% inhibition, respectively, at a dosage of 5 mg/20 g mouse. Ethyl acetate extract also showed hypoglycemic activity more effectively than the other extracts by providing a 56.7% reduction in blood glucose level.

## 8. Clinical Studies

Health-promoting effects of *Senna* and its other species have been evaluated by a large number of researchers around the world while clinical trials have been conducted in limited cases (Table 4). Therefore, in this section, we are presenting quantified data on *Senna* and its clinical trials (previous and latest).

Mcnicol [225] performed a clinical experiment to evaluate the activity of tablets prepared using Senna (standardized preparation) on human bowel function and its possible side effects. The experiment was carried out in two phases: (a) first is the administration of the drug to 52 ward patients; (b) the drug was administered to 126 volunteer medical students. The *Senna* tablets were prepared in two different batches. The results demonstrated that the mean values for “speed of action” of *Senna* preparation (3 tablets) were recorded as 9.7 hours with ward patients and 12.15 hours among student volunteers, respectively. The frequency of griping, looseness of stool, and multiple bowel movements in ward patients have been recorded in dose-dependent patterns (increased with rising dosage). In addition, results confirmed that there is no significant difference between male and female responses. Thamlikitkul et al. [226] performed a randomized controlled experiment to evaluate the efficacy of *S. alata* against constipation. A total of 80 candidates participated in this study, and the differences observed between both groups (placebo & mist. Alba; and placebo & *S. alata*) were statistically highly significant (*p* < 0.001).

Kinnunen et al. [227] evaluated the safety and efficacy profile of laxatives containing *Senna* in treating constipation patients using lactulose as standard medication. The present study was carried out in a total of 30 patients (mainly bed-ridden due to degenerative diseases, age: 65-94 years). One week run-in without laxatives was followed by 5 weeks (a) of a daily dose of 14.8 mg (20 mL) laxative plus *Senna* or 20.1 mg (30 mL) lactulose and (b) crossed medicines (5-week period). The results indicated that bulk laxative plus *Senna* (14.8 mg dose) when given daily resulted in significantly (*p* < 0.005) more frequent bowel habits (4.5 vs. 2.2-19/week) compared to that of lactulose (daily dose of 14.8 mg). In other words, bulk laxative plus *Senna* produced efficiently treated constipation patients.

Damodaran and Venkataraman [228] from India reported the therapeutic effectiveness of *S. alata* leaves against *Pityriasis versicolor* in humans. The study was completed among 200 candidates (age: 16-60 years) of Tamil Nadu (Indian State) within 10 years. Different concentrations of plant extract (80%, 90%, and 100%) were used at affected areas (trunk, neck, hands, and face) of the body. The results indicated that *S. alata* leaf extract could be employed as a herbal remedy having no side effects, for curing *P. versicolor*.

Ramesh et al. [229] carried out a controlled comparative study of *Misrakasneham* (Ayurvedic formulation) and laxative *Senna* tablets (purified *Senna* extract) against opioid-induced constipation. *Misrakasneham* (a combination of 21 different types of herbs, castor oil, purified butter, and milk) is a centuries-old Ayurvedic medicine. The present study was conducted in 50 patients with advanced cancer aged 15 years and categorized into two groups (25 each). The first group received *Misrakasneham* while the second group received *Senna* tablets in three steps during the 14-day study. The results demonstrated that 85% of the *Misrakasneham* group and 69% of the laxative *Senna* group had satisfactory bowel movements with no statistical difference (*p* > 0.2). In addition, *Misrakasneham* data showed interesting results in terms of efficacy and was recommended as a possible candidate for opioid-induced constipation.

van Gorkom et al. [230] reported the effects of sennosides on histology of colonic mucosa and bowel preparation. In this experiment, a total of 171 candidates participated who were further split into two groups: (a) *n* = 84 candidates treated with 1 mL/kg of a syrup containing 2 mg/mL sennosides A and B and 3-5 L of a lavage solution and (b) *n* = 87 candidates treated with 3-5 L of lavage solution. The results demonstrated that both groups showed no difference in tolerance or quality of bowel preparation. In addition, group a (10/19) also showed a rapid increase of mononuclear infiltrate in the lamina propria compared to group b (2/21), respectively (*p* = 0.0005).

## 9. Safety and Side Effects

In traditional medicine, the leaves of *Senna* traditionally are used as laxatives in the form of pellets prepared with dried figs and plums. The anthraquinone laxatives like *Senna* are extremely useful drugs, but appropriate usage is highly important, although most of the reported side effects are mild and transit.

**Figure 5:**
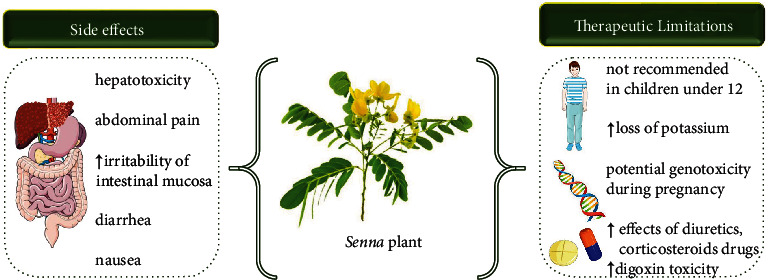
Summarized scheme with side effects and clinical therapeutic limitations of *Senna* plant.

*Senna* is generally safe and well tolerated but can cause adverse events when it is used in high doses and for a long period (Figure 5). Most of the adverse effects are mild and transient. The liver injury, including hepatotoxicity, has been reported in several case studies when *Senna* has been used prolonged, and the symptoms were mild-to-moderate in severity and solved rapidly with discontinuation [243–245]. In all cases, the correlation between side effects was explained by abuse of Senna in laxative purpose.

Derivatives of sennosides present in the leaves and pods may affect increasing irritability on the intestinal mucosa, which could cause abdominal pain and spasm in a sensitive person. It can also lead to diarrhoea, intensification of menstrual bleeding, and dark urine. It is recommended to take herbal tea or capsules/tablets/syrup of *Senna* in the evening before sleep, as effects start 6 to 12 hours later. Also, drugs that contain *Senna* are available in the form of rectal suppositories.

The prolonged use of *Senna* causing the spasm is the sign that it is necessary to stop future taken. In rare cases, vomiting and nausea may occur. Chronic use of *Senna* and other laxative herbs leads to increased potassium excretion, resulting in spasms, muscle weakness, and heart failure. However, in very well-explained patient conditions, these types of herbal drugs should be avoided. However, the full safety profile of these herbals is controversial like the opponent attitude of FDA and EMA regarding their consumption in some vulnerable groups of people.

## 10. Therapeutic Perspectives and Clinical Gaps

Traditional and modern medicines, in case of decreasing the intestine motility, take into consideration two classes of curative substances: drugs that increase the volume of the gut contents and facilitate mass flow [246, 247]. Among herbal substances, here, we have substances rich in sugars (dried plums and figs) and herbals with mucus, such as flax seeds. Another approach is medicines that contain substances that have a mild irritant effect on the intestinal mucosa to promote intestinal motility [248, 249]. Among these remedies are the species from the genus *Senna*.

Genus *Senna* is well recognized as the most used laxative herbal treatment, also available without a prescription. Even before we knew its composition, *Senna* was used for centuries in phytotherapy for the same purpose. The main type of *Senna* genus used in medicine is *S. alexandrina*, known in commerce as Alexandria Senna, and Tinnevelly *Senna* [250]. *Senna* plants are widely used herbal medicine in the treatment of functional constipation. As the beneficial parts of the plant in phytotherapy, both the mature pods and the dried leaves are used. They contain natural chemical compounds, called anthraquinone, which are glycoside derivatives of anthracene, and the major compounds are sennosides A and B, which are available in the market [251]. The sennosides A and B have been broken down by the bacterial flora in the colon and result in the production of the main active metabolites rhein and rheinanthrone [252]. The working of anthraquinones includes the hindrance of NaCl absorption in the colon and the stimulation of Cl secretion, by inhibiting the (Na^+^, K^+^)-ATPase [253].

Additionally, *S. alexandrina* is used in case of bowel irritable colon, as a pretreatment before diagnostic tests like colonoscopy [254] and as a supplement for weight reduction [255]. While the treatment with active compounds from genus *Senna* is widely used in different laxative drugs taken orally in liquid or solid dosage forms, in the form of instant tea and herbal tea, however, there are controversies in their usage.

European Medical Agency (EMA) reference the use of *Senna* [256] only in cases of periodical constipation, while long term is not recommended due to acute dehydration which is followed by loss of electrolytes. Also, EMA do not recommend *Senna* in case of pregnancy, breast feeding, dehydration, different forms of intestinal obstructions, ulcers, and ulcerative colitis, inflammatory bowel disease including Crohn's disease, pain and spasm in stomach, unknown etiology, and rectal bleeding.

EMA does not recommend using the *Senna* as a laxative treatment in children under 12, but off-label use has been reported (Figure 5). On the other hand, the USA Food and Drug Administration (FDA) prescribes 17.2 mg (7.5 to 30 mg) per day for people 12 years and older and 8.5 mg for children under 12 and allowing the usage of botanical laxatives containing Senna in children under 12. Based on a recently published review on *Senna* side effects as a long-term therapy in children by Vilanova-Sanchez et al. [257], *Senna* can be a safely employed option in treating functional constipation in children. However, more evidences are needed to confirm this conclusion and to change the attitude of EMA, who recently revised the herbal monography of *Senna* still stated that *Senna* is not recommended for children under 12 years [256].

Although some researches of *Senna* have found that it is effective in a short-term usage of constipation treatment in pregnancy [258–260] and does not have the teratogenic potential [261], intake of *Senna* during the pregnancy is allowed only in some countries like the USA.

*Senna* is still contraindicated by EMA recommendation because of experimental data that indicated possibly a genotoxic risk of several anthranoids, e.g., emodin and aloe-emodin [262]. While the use of *Senna* in breastfeeding women is not recommended, there is evidence that anthraquinone drugs in lactating mothers do not carry a risk of producing a laxative effect in the infant [263–265]. However, there are available data from other studies in which laxative effect on the bowels was observed in infants [258]. Despite controversial findings, still, the official recommendation is to avoid the use of it.

*Senna* should not be used for a longer period, no longer than 1-2 weeks, nor with medicines that lead to loss of potassium (diuretics, cardiotonic drugs, and corticosteroids). The caution should be exercised when used with antiarrhythmic and cardiotonic drugs and medicinal products inducing QT-prolongation, as it may potentiate their effect. All of these effects are correlated with hypokalemia [266, 267]. It has been found that usage of sennosides and digoxin in combination is linked with a modestly increased risk of digoxin toxicity in heart failure patients [268].

Particular attention, based on the animal studies, should be exerted in the patients with kidney and liver disorders during chronic use of *Senna*-based products [269]. Additionally, studies performed on rats showed that long-term administration of extracts of *Senna* does not promote gastrointestinal, liver, kidney, or adrenal tumors in the rats [270–272].

## 11. Concluding Remarks

This review showed that various parts of the *Senna* plant such as roots, stem, leaves, and seeds are traditionally used to treat many ailments and its extract has antioxidant, antimicrobial, and important health-promoting activities. These biological activities are attributed to the many phytochemicals contained in the genus Senna. Epicatechin, proanthocyanidins, scutellarein, rutin, and sennoides are just a few bioactive compounds of the genus *Senna* that are responsible for their bioactivity. Numerous studies *in vitro* and *in vivo* have been performed to establish the anti-infective and antioxidant properties of *Senna* extracts. Studies on the consumption of *Senna* over a period have shown that *Senna* is safe, but chronic use has adverse and limiting effects in medical practice. Among them, the laxative disease is a condition related to the massive use of *Senna-*based laxatives with an increased loss of potassium ions and the possibility of interaction with other drugs prescribed for heart disease. Based on the analysis of the studies selected in the study, this review opens new therapeutic perspectives of the *Senna* plant for antioxidant and especially anti-infective effects in the digestive tract.

## Figures and Tables

**Table 1:** Traditional and folk medical usage of *Senna* species.

*Senna species*	Country/culture	Part of plant	Internal usage	External usage	Ref
*Senna alata* (L.) Roxb.	Bangladesh	Leaves	Helminthiasis	Ringworm, eczema	[37, 38]
	Benin Republic	Whole plant	Diabetes	—	[12]
	Bolivia	Root, leaves	Malaria, salmonella, fever, cold	Bath	[39]
	Brazil	Root, whole plant, flower, leaves	Flu, cough, malaria	Ringworms, scabies, blotch, eczema, tinea infections	[12, 40]
	Cameroon	Stem, bark, leaves	Gastroenteritis, hepatitis	Ringworm, dermal infections	[12]
	China	Stem, bark, leaves seed, root, leaves, flower, whole plant	Intestinal parasitosis, helminthiasis, diabetes, uterus disorder, asthma, constipation, fungal infections, poor eyesight diabetes	—	[12]
	Cuba	Whole plant	Diabetes	—	[41]
	Egypt	Leaves	Constipation	—	[12]
	Ghana	Whole plant	Diabetes	—	[12]
	Guatemala	Whole plant flower, leaves	Flu, malaria	Ringworms, tinea infections scabies, eczema, blotch	[12]
	Guinea	Whole plant flower, leaves	Flu, malaria	Ringworms, scabies, blotch, eczema, tine infections	[12]
	India	Stem, bark, leaves, seed, root leaves, flower the whole plant, leaves	Diabetes, hemorrhoids, inguinal hernia, intestinal parasitosis, syphilis, uterus disorder, helminthiasis constipation, fungal infection diabetes	Skin diseases, ringworm	[12, 42]
	Nigeria	Stem, leaves, root whole plant	Constipation, diarrhoea, respiratory tract infection, body and abdominal pain, stress, convulsion, diabetes	Wound, skin diseases, burns, toothache, dermal infections	[12]
	Philippines	Stem, bark, leaves seed, root leaves, flower leaves	Hemorrhoids, inguinal hernia, syphilis, intestinal parasitosis, diabetes, uterus disorder, helminthiasis, constipation, fungal infections	Skin diseases, wound	[12, 43]
	Sierra Leone	Leaves	Abortion pain, facilitate delivery	—	[12]
	Thailand	Leaves	Constipation, flatulence, inflammation	Abscesses, wounds, ringworm, itching	[33, 44]
	Togo	Whole plant	Diabetes	—	[12]

*Senna alexandrina* Mill.	Cyprus	Fruit	Constipation	—	[45]
	Djibouti	Leaves	Constipation, injuries	Skin diseases	[46]
	Egypt	Leaves	Constipation	—	[47]
	Pakistan	Leaves, pod	Constipation, rheumatism, backache, asthma, anaemia typhoid fever, jaundice, pneumonia, leprosy	Wound, pimples	[48]
	Qatar	Leaves	Constipation, stomach cramps	—	[49]
	Sudan	Leaves, fruits	Constipation, git-disorders	—	[50]
	Thailand	Leaf pod	Constipation stomach pain	—	[33]
	UAE	Leaves	Constipation, stomach cramps	—	[49]

*Senna auriculata* (L.) Roxb.	India	Flower leaves	Diabetes	—	[51]

*Senna didymobotrya* (Fresen.) H.S.Irwin & Barneby	South Africa	Leaves	Blood coagulation	—	[52]

*Senna fruticosa* (Mill.) H.S.Irwin & Barneby	Panama	Stem, leaves	—	Body ache	[53]

*Senna garrettiana* (Craib) H.S.Irwin & Barneby	Thailand	Heartwood	Constipation, cough, emmenagogue	—	[33]

*Senna hirsuta* (L.) H.S.Irwin & Barneby	Thailand	Debarked stem	Fever, muscle spasm, poisoning, drunkenness	—	[33, 44]

*Senna italica* Mill.	Bahrain	Leaves, seed	Constipation, stomach cramps	—	[49]
	Djibouti	Leaves	Constipation	—	[46]
	Egypt	Leaves	Constipation, bacterial infection, tumors	—	[47]
	Iran	Leaves	Constipation, obesity, hemorrhoids	—	[54]
	Pakistan	Leaves	Backache joints pain, headache, migraine	—	[55]
	Qatar	Leaves, seed	Constipation, stomach cramps	—	[49]
	Saudi Arabia	Leaves, seed	Constipation, stomach cramps	—	[49]
	UAE	Leaves, seed	Constipation, stomach cramps	—	[49]

*Senna multiglandulosa* (Jacq.) H.S.Irwin & Barneby	Peru	Not specified	—	Wound disinfectant agent	[56]

*Senna occidentalis* (L.) Link	Bolivia	Root, seed	Dysentery	Bath, ringworm	[39]
	Cuba	Not specified	Liver pain, rheumatism, arthrosis, catarrh, muscular pain, hemorrhoids, pneumonia, venereal diseases, impotence	—	[41]
	Guatemala	Leaves, aerial part	Fever, measles, chickenpox	—	[57]
	India	Leaves, root seed	Respiratory diseases, cough, constipation, malaria, diabetes, indigestion, urinary disorder	Skin problems, skin disorders, pimples	[42, 58, 59]
	Tanzania	Root	Spasms, malaria, helminthiasis	—	[60]
	Thailand	Leaves, fruit	Diarrhoea	—	[44]
	Uganda	Leaves	Malaria	—	[35]

*Senna petersiana* (*Bolle*) *Lock*	Eastern Africa	Not specified	Flatulence	—	[61]
	Tropical Africa	Not specified	Constipation, gonorrhoea	—	[61]
	South Africa	Seed	Venereal diseases, infertility constipation, gonorrhoea	—	[61, 62]

*Senna siamea* (Lam.) H.S.Irwin & Barneby	Thailand	Leaves, flower	Constipation, insomnia hypertension	—	[33, 44]

*Senna singueana* (Delile) Lock	Sudan	Root	Constipation	—	[63]
	Tanzania	Root	Diabetes	—	[64]

*Senna sophera* (L.) Roxb.	Bangladesh	Leaves root	Dyspepsia, asthma, bronchitis, hiccup, gonorrhoea dyspepsia	—	[37, 38, 65]
	India	Bark	Respiratory disorders	—	[42]

*Senna timoriensis* (DC.) H.S.Irwin & Barneby	Thailand	Heartwood	Stimulate menstruation	—	[33]

*Senna tora* (L.) Roxb.	China	Not specified	Stomach disorders, liver diseases, poor eyesight, weakness, diuretic	—	[66]
	Thailand	Seed leaves	Constipation, urethral stones, diuretic, constipation, insomnia	—	[33, 44]
	India	Seed leaves		Rheumatic swelling and pain, skin diseases	[42, 67]

*Senna uniflora* (Mill.) H.S.Irwin & Barneby	Cuba	Not specified	Bleeding, rheumatism, arthrosis	—	[41]

**Table 2:** Summary of several *in vitro* studies about the antioxidant activity and total phenolic content of genus *Senna.*

*Senna genus*	Part of plant—solvent—procedure (if any)	Method	Result						References
*Senna gardneri* (Benth.) H.S.Irwin & Barneby *Senna macranthera* (Collad.) H.S.Irwin & Barneby *Senna splendida* (Vogel) H.S.Irwin & Barneby *Senna trachypus* (Benth.) H.S.Irwin & Barneby	Root, leaves—ethanol	DPPH, ABTS—Folin-Ciocalteu		DPPH (IC_50_ mg/mL)		ABTS (TEAC)		TPC (mg GAE/100 g)	[188]
			Root = Sg	0,396		57,13		214,25	
			Sm	0,534		53,72		122,09	
			Ss	0,502		36,02		146,60	
			St	0,253		64,47		1277,34	
			Leaves = Sg	0,089		47,91		338,76	
			Sm	0,424		26,72		207,71	
			Ss	0,286		29,63		148,24	
			St	0,401		30,71		322,09	
*Senna velutina* (Vogel) H.S.Irwin & Barneby	Leaves—ethanol	DPPH							[185]
*Senna reticulata* (Willd.) H.S.Irwin & Barneby	Aerial parts—Methyl tert-butyl ether (MTBE)/methanol (90 : 10)	DPPH, ORAC-Folin-Ciocalteu	DPPH (𝜇g/mL)			ORAC (mmol TE/mg extract)		TPC (mg GAE/g)	[193]
			72.90			2.68		79.3	
*Senna alata* (L.) Roxb.	Roots—acetone, ethanol, water	DPPH, ABTS (IC50)		DPPH (𝜇g/mL)		ABTS (mmol TE/mg extract)		TPC (mg GAE/g)	[189]
			Acetone	82.42		64,93		21,42	
			Ethanol	45,18		39,14		78,21	
			Water	61,15		48,3		46,3	
*Senna bicapsularis* (L.) Roxb.	Flowers—ethanol, water	DPPH, FRAP—Folin-Ciocalteu	% DPPH inhibition			FRAP (𝜇moles Fe(II)/100 g).		TPC (mg GAE/100 g)	[194]
			Ethanol	99,51		2403.15		26223.78	
			Water	96,51		1966.30		9468.18	
*Senna italica* mill.	Aerial parts—ethyl acetate, n-butanol	ABTS	% Inhibition						[88]
			Ethyl acetate					82,9	
			n-Butanol					85,7	
*Senna siamea* (Lam.) H.S.Irwin & Barneby	Leaves—ethanol (49%)—ultrasound-assisted (UA)	DPPH, FRAP—Folin-Ciocalteu		%DPPH inhibition.		FRAP (mM FeSO4/g)		TPC (mg GAE/g)	[190]
			Ethanol	80,49		8,08		455,42	
			UA	91,83		11,41		575,23	
*Senna alexandrina* Mill.	Flowers, leaves—ethanol (70%)—microwave, Soxhlet, marination, reflux and sonication	DPPH—HPLC-ESI-MS/MS		%Inhibition (IC50) = microwave	Soxhlet	Marination	Reflux	Sonication	[191]
			Flowers	3,1	3,4	3,5	6,5	5,9	
			Leaves	3,6	4,2	5,6	6,2	7,4	
*Senna alata* (L.) Roxb.	Leaves—ethanol	Chemiluminescence measurement							[192]
*Senna alata* (L.) Roxb.	Root, stem, seed, leaves and flower—methanol/water (80%)	FRAP-Folin-Ciocalteu			FRAP (g/100 g)		TPC (g/100 g)		[195]
			Root		0,255		1,69		
			Stem		0,457		2,27		
			Seed		0,345		2,59		
			Leave		0,560		2,33		
			Flower		0,565		1,36		

**Table 3:** Anti-infectious activity of genus *Senna.*

Effect	Microorganism	Antimicrobial assay	*Senna* genus	Plant part-solvent	Result-solvent	References
Antiprotozoal	*Haemonchus contortus*	Effective dose determination for ED50	*Senna occidentalis*	Crude plant-aqueous extract	0.13 mg/mL	[221]
	*Haemonchus contortus*	Effective dose determination for ED50	*Senna occidentals*	Crude plant-hydroalcoholic extract	0.17 mg/mL	[221]
	*Schistosoma mansoni*	Effective dose determination for ED50	*Senna spectabilis*	Flower-ethanol extract	495.4 *μ*g/mL	[201]

Antibacterial	*Bacillus cereus*	Diameter of the inhibition zone	*Senna alexandrina*	Leaves-methanol	11.0 mm	[211]
	*Bacillus cereus*	Diameter of the inhibition zone	*Senna alexandrina*	Leaves-infusion	10.0 mm	[211]
	*Bacillus cereus*	Diameter of the inhibition zone	*Senna alexandrina*	Leaves-decoction	ND	[211]
	*Bacillus cereus*	Diameter of the inhibition zone	*Senna alexandrina*	Leaves-hydrosol	ND	[211]
	*Bacillus cereus*	Agar disk diffusion method, zone of inhibition	*Senna bicapsularis*	Flower-ethanol extract	7 mm	[194]
	*Bacillus cereus*	Agar disk diffusion method, zone of inhibition	*Senna bicapsularis*	Flower-distilled water	8 mm	[194]
	*Bacillus cereus*	Paper disk diffusion method, MIC	*Senna siamea*	Leaf ethanol/water mixture extract	300 mg/mL	[190]
	*Bacillus subtilis*	Disc agar technique, inhibition zone	*Senna italica*	Aerial part-n-butanol extract	9.3 mm	[88]
	*Bacillus subtilis*	Disc agar technique, inhibition zone	*Senna italica*	Aerial part-ethyl acetate extract	14 mm	[88]
	*Candida albicans*	Disc agar technique, inhibition zone	*Senna italica*	Aerial part-n-butanol extract	12 mm	[88]
	*Candida albicans*	Disc agar technique, inhibition zone	*Senna italica*	Aerial part-ethyl acetate extract	6 mm	[88]
	*Enterobacter aerogenes*	Disc agar technique, inhibition zone	*Senna italica*	Aerial part-n-butanol extract	12.4 mm	[88]
	*Enterobacter aerogenes*	Disc agar technique, inhibition zone	*Senna italica*	Aerial part-ethyl acetate extract	9 mm	[88]
	*Erwinia* spp.	Disc agar technique, inhibition zone	*Senna italica*	Aerial part-n-butanol extract	10 mm	[88]
	*Erwinia* spp.	Disc agar technique, inhibition zone	*Senna italica*	Aerial part-ethyl acetate extract	8 mm	[88]
	*Erwinia chrysanthemi*	Agar well diffusion	*Senna spectabilis*	Leaf-dichloromethane Leaf-methanol	12.00 ± 1.70 *mm* 13.00 ± 2.10 *mm*	[223]
	*Erwinia chrysanthemi*	Agar well diffusion	*Senna spectabilis*	Flower-dichloromethane Flower-methanol	9.70 ± 0.60 mm 10.00 ± 2.50 mm	[223]
	*Erwinia chrysanthemi*	Agar well diffusion	*Senna spectabilis*	Stem-dichloromethane Stem-methanol	9.30 ± 1.20 mm 16.00 ± 1.20 mm	[223]
	*Escherichia coli*	Agar well diffusion	*Senna alata*	Leaf-ethanol	17.2 ± 0.3 mm	[203]
	*Escherichia coli*	Agar well diffusion	*Senna alata*	Leaf-water	10.2 ± 0.2 mm	[203]
	*Escherichia coli*	Inhibition zone (filter paper disc diffusion method)	*Senna occidentalis*	Whole plant ethanol extract	7-8 mm	[212]
	*Escherichia coli*	Disc agar technique, inhibition zone	*Senna italica*	Aerial part-n-butanol extract	19 mm	[88]
	*Escherichia coli*	Disc agar technique, inhibition zone	*Senna italica*	Aerial part-ethyl acetate extract	16 mm	[88]
	*Escherichia coli*	The cup plate agar diffusion method	*Senna alata*	Leaf hot water/leaf-methanol/leaf-acetone	3 mm/4 mm/3 mm	[205]
	*Escherichia coli*	The cup plate agar diffusion method	*Senna alata*	Root hot water/root-methanol/root-acetone	4 mm/4 mm/3 mm	[205]
	*Escherichia coli*	Minimum inhibitory concentration	*Senna alata*	Leaf-methanol/root-methanol	8 mg/mL/6 mg/mL	[205]
	*Escherichia coli*	Minimum microbicidal concentration	*Senna alata*	Leaf-methanol/root-methanol	8 mg/mL/6 mg/mL	[205]
	*Klebsiella aerogenes*	Inhibition zone (filter paper disc diffusion method)	*Senna occidentalis*	Whole plant ethanol extract	ND	[212]
	*Klebsiella pneumoniae*	Agar disk diffusion method, zone of inhibition	*Senna bicapsularis*	Flower-ethanol extract	7 mm	[194]
	*Klebsiella pneumoniae*	Agar disk diffusion method, zone of inhibition	*Senna bicapsularis*	Flower-distilled water	9 mm	[194]
	*Listeria monocytogenes*	Agar disk diffusion method, zone of inhibition	*Senna bicapsularis*	Flower-ethanol extract	ND	[194]
	*Listeria monocytogenes*	Agar disk diffusion method, zone of inhibition	*Senna bicapsularis*	Flower-distilled water	ND	[194]
	*Neisseria gonorrhoeae*	Minimum inhibitory concentration	*Senna podocarpa*	Root hydroethanol extract	100 to 400 mg/L	[113]
	*Propionibacterium acnes*	Disc diffusion assay, minimum inhibitory concentration	*Senna alata*	Crude plant extract	0.625 mg/mL	[224]
	*Propionibacterium acnes*	Disc diffusion assay, minimum inhibitory concentration	*Senna occidentalis*	Crude plant extract	2.5 mg/mL	[224]
	*Propionibacterium acnes*	Disc diffusion assay, minimum inhibitory concentration	*Senna siamea*	Crude plant extract	1.25 mg/mL	[224]
	*Proteus mirabilis*	The cup plate agar diffusion method	*Senna alata*	Leaf hot water/leaf-methanol/leaf-acetone	2 mm/3 mm/2 mm	[205]
	*Proteus mirabilis*	The cup plate agar diffusion method	*Senna alata*	Root hot water/root-methanol/root-acetone	3 mm/3 mm/2 mm	[205]
	*Proteus mirabilis*	Minimum inhibitory concentration	*Senna alata*	Leaf-methanol/root-methanol	10 mg/mL/8 mg/mL	[205]
	*Proteus mirabilis*	Minimum microbicidal concentration	*Senna alata*	Leaf-methanol/root-methanol	10 mg/mL/6 mg/mL	[205]
	*Proteus vulgaris*	Inhibition zone (filter paper disc diffusion method)	*Senna occidentalis*	Whole plant ethanol extract	7-10 mm	[212]
	*Pseudomonas aeruginosa*	The cup plate agar diffusion method	*Senna alata*	Leaf hot water/leaf-methanol/leaf-acetone	3 mm/3 mm/3 mm	[205]
	*Pseudomonas aeruginosa*	The cup plate agar diffusion method	*Senna alata*	Root hot water/root-methanol/root-acetone	3 mm/3 mm/3 mm	[205]
	*Pseudomonas aeruginosa*	Minimum inhibitory concentration	*Senna alata*	Leaf-methanol/root-methanol	10 mg/mL/8 mg/mL	[205]
	*Pseudomonas aeruginosa*	Minimum microbicidal concentration	*Senna alata*	Leaf-methanol/root-methanol	10 mg/mL/8 mg/mL	[205]
	*Pseudomonas aeruginosa*	Paper disk diffusion method, MIC	*Senna siamea*	Leaf ethanol/water mixture extract	300 mg/mL	[190]
	*Pseudomonas aeruginosa*	Diameter of the inhibition zone	*Senna alexandrina*	Leaves-methanol	9.0 mm	[211]
	*Salmonella typhimurium*	Agar well diffusion	*Senna alata*	Leaf-ethanol	12.1 ± 0.1 *mm*	[203]
	*Salmonella typhimurium*	Agar well diffusion	*Senna alata*	Leaf-water	10.1 ± 0.1 *mm*	[203]
	*Salmonella typhimurium*	The cup plate agar diffusion method	*Senna alata*	Leaf hot water/leaf-methanol/leaf-acetone	3 mm/4 mm/4 mm	[205]
	*Salmonella typhimurium*	The cup plate agar diffusion method	*Senna alata*	Root hot water/root-methanol/root-acetone	3 mm/4 mm/4 mm	[205]
	*Salmonella typhimurium*	Minimum inhibitory concentration	*Senna alata*	Leaf-methanol/root-methanol	8 mg/mL/6 mg/mL	[205]
	*Salmonella typhimurium*	Minimum microbicidal concentration	*Senna alata*	Leaf-methanol/root-methanol	6 mg/mL/8 mg/mL	[205]
	*Salmonella typhimurium*	Paper disk diffusion method, MIC	*Senna siamea*	Leaf ethanol/water mixture extract	300 mg/mL	[190]
	*Shigella* spp.	Disc agar technique, inhibition zone	*Senna italica*	Aerial part-n-butanol extract	7.8 mm	[88]
	*Shigella* spp.	Disc agar technique, inhibition zone	*Senna italica*	Aerial part-ethyl acetate extract	8.6 mm	[88]
	*Shigella flexneri*	The cup plate agar diffusion method	*Senna alata*	Leaf hot water/leaf-methanol/leaf-acetone	4 mm/4 mm/4 mm	[205]
	*Shigella flexneri*	The cup plate agar diffusion method	*Senna alata*	Root hot water/root-methanol/root-acetone	3 mm/4 mm/3 mm	[205]
	*Shigella flexneri*	Minimum inhibitory concentration	*Senna alata*	Leaf-methanol/root-methanol	8 mg/mL/5 mg/mL	[205]
	*Shigella flexneri*	Minimum microbicidal concentration	*Senna alata*	Leaf-methanol/root-methanol	6 mg/mL/5 mg/mL	[205]
	*Staphylococcus aureus*	Inhibition zone (filter paper disc diffusion method)	*Senna occidentalis*	Whole plant ethanol extract	8-9 mm	[212]
	*Staphylococcus aureus*	Agar disk diffusion method, zone of inhibition	*Senna bicapsularis*	Flower-ethanol extract	ND	[194]
	*Staphylococcus aureus*	Agar disk diffusion method, zone of inhibition	*Senna bicapsularis*	Flower-distilled water	7 mm	[194]
	*Staphylococcus aureus*	Agar well diffusion	*Senna alata*	Leaf-ethanol	20.1 ± 0.1 mm	[203]
	*Staphylococcus aureus*	Agar well diffusion	*Senna alata*	Leaf-water	18.2 ± 0.3 mm	[203]
	*Staphylococcus aureus*	Disc agar technique, inhibition zone	*Senna italica*	Aerial part-n-butanol extract	11 mm	[88]
	*Staphylococcus aureus*	Disc agar technique, inhibition zone	*Senna italica*	Aerial part-ethyl acetate extract	6 mm	[88]
	*Staphylococcus aureus*	The cup plate agar diffusion method	*Senna alata*	Leaf hot water/leaf-methanol/leaf-acetone	5 mm/5 mm/5 mm	[205]
	*Staphylococcus aureus*	The cup plate agar diffusion method	*Senna alata*	Root hot water/root-methanol/root-acetone	4 mm/4 mm/4 mm	[205]
	*Staphylococcus aureus*	Minimum inhibitory concentration	*Senna alata*	Leaf-methanol/root-methanol	6 mg/mL/5 mg/mL	[205]
	*Staphylococcus aureus*	Minimum microbicidal concentration	*Senna alata*	Leaf-methanol/root-methanol	6 mg/mL/5 mg/mL	[205]
	*Staphylococcus epidermidis*	Disc diffusion assay, minimum inhibitory concentration	*Senna alata*	Crude plant extract	2.5 mg/mL	[224]
	*Staphylococcus epidermidis*	Disc diffusion assay, minimum inhibitory concentration	*Senna occidentalis*	Crude plant extract	>5 mg/mL	[224]
	*Staphylococcus epidermidis*	Disc diffusion assay, minimum inhibitory concentration	*Senna siamea*	Crude plant extract	>5 mg/mL	[224]
	*Streptococcus pyogenes*	The cup plate agar diffusion method	*Senna alata*	Leaf hot water/leaf-methanol/leaf-acetone	6 mm/6 mm/5 mm	[205]
	*Streptococcus pyogenes*	The cup plate agar diffusion method	*Senna alata*	Root hot water/root-methanol/root-acetone	5 mm/6 mm/5 mm	[205]
	*Streptococcus pyogenes*	Minimum inhibitory concentration	*Senna alata*	Leaf-methanol/root-methanol	6 mg/mL/3 mg/mL	[205]
	*Streptococcus pyogenes*	Minimum microbicidal concentration	*Senna alata*	Leaf-methanol/root-methanol	6 mg/mL/3 mg/mL	[205]
	*Xanthomonas axonopodis*	Agar well diffusion	*Senna spectabilis*	Leaf-dichloromethane leaf-methanol	9.70 ± 0.60 mm 110.0 ± 0.60 mm	[223]
	*Xanthomonas axonopodis*	Agar well diffusion	*Senna spectabilis*	Flower-dichloromethane flower-methanol	11.00 ± 1.20 mm 14.00 ± 3.50 mm	[223]
	*Xanthomonas axonopodis*	Agar well diffusion	*Senna spectabilis*	Stem-dichloromethane stem-methanol	12.00 ± 2.60 mm 25.00 ± 50.0 mm	[223]

Antifungal	*Aspergillus flavus*	Inhibition zone (filter paper disc diffusion method)	*Senna occidentalis*	Whole plant ethanol extr80t	12-30 mm	[212]
	*Aspergillus flavus*	Agar well diffusion	*Senna alata*	Leaf-ethanol	22.1 ± 0.1 *mm*	[203]
	*Aspergillus flavus*	Agar well diffusion	*Senna alata*	Leaf-water	20.1 ± 0.1 *mm*	[203]
	*Aspergillus flavus*	The cup plate agar diffusion method	*Senna alata*	Leaf hot water/leaf-methanol/leaf-acetone	2 mm/3 mm/2 mm	[205]
	*Aspergillus flavus*	The cup plate agar diffusion method	*Senna alata*	Root hot water/root-methanol/root-acetone	2 mm/3 mm/2 mm	[205]
	*Aspergillus flavus*	Minimum inhibitory concentration	*Senna alata*	Leaf-methanol/root-methanol	50 mg/mL/50 mg/mL	[205]
	*Aspergillus flavus*	Minimum microbicidal concentration	*Senna alata*	Leaf-methanol/root-methanol	50 mg/mL/50 mg/mL	[205]
	*Aspergillus niger*	Inhibition zone (filter paper disc diffusion method)	*Senna occidentalis*	Whole plant ethanol extract	14-22 mm	[212]
	*Aspergillus niger*	Agar well diffusion	*Senna alata*	Leaf-ethanol	25.2 ± 0.3 *mm*	[203]
	*Aspergillus niger*	Agar well diffusion	*Senna alata*	Leaf-water	27.2 ± 0.2 *mm*	[203]
	*Aspergillus niger*	Cup-plate method, mean zone of inhibition	*Senna alata*	Ethanolic leaf extract	17.6-25.8 mm	[207]
	*Aspergillus niger*	Cup-plate method, mean zone of inhibition	*Senna alata*	Aqueous leaf extracts	10.5-33.8 mm	[207]
	*Aspergillus niger*	The cup plate agar diffusion method	*Senna alata*	Leaf hot water/leaf-methanol/leaf-acetone	2 mm/3 mm/2 mm	[205]
	*Aspergillus niger*	The cup plate agar diffusion method	*Senna alata*	Root hot water/root-methanol/root-acetone	2 mm/3 mm/3 mm	[205]
	*Aspergillus niger*	Minimum inhibitory concentration	*Senna alata*	Leaf-methanol/root-methanol	50 mg/mL/50 mg/mL	[205]
	*Aspergillus niger*	Minimum microbicidal concentration	*Senna alata*	Leaf-methanol/root-methanol	50 mg/mL/50 mg/mL	[205]
	*Candida albicans*	Agar well diffusion	*Senna alata*	Leaf-ethanol	18.2 ± 0.2 *mm*	[203]
	*Candida albicans*	Agar well diffusion	*Senna alata*	Leaf-water	14.1 ± 0.1 *mm*	[203]
	*Candida albicans*	Cup-plate method, mean zone of inhibition	*Senna alata*	Ethanolic leaf extract	19.8-36 mm	[207]
	*Candida albicans*	Cup-plate method, mean zone of inhibition	*Senna alata*	Aqueous leaf extracts	20.2-30.0 mm	[207]
	*Candida albicans*	Agar cup method, clearing zone	*Senna alata*	Leaf-chloroform extract	ND	[222]
	*Candida albicans*	Agar cup method, clearing zone	*Senna alata*	Leaf-ethyl acetate extract	15-20 mm	[222]
	*Candida albicans*	The cup plate agar diffusion method	*Senna alata*	Leaf hot water/leaf-methanol/leaf-acetone	2 mm/4 mm/3 mm	[205]
	*Candida albicans*	The cup plate agar diffusion method	*Senna alata*	Root hot water/root-methanol/root-acetone	3 mm/4 mm/4 mm	[205]
	*Candida albicans*	Minimum inhibitory concentration	*Senna alata*	Leaf-methanol/root-methanol	35 mg/mL/25 mg/mL	[205]
	*Candida albicans*	Minimum microbicidal concentration	*Senna alata*	Leaf-methanol/root-methanol	25 mg/mL/25 mg/mL	[205]
	*Candida albicans*	Agar cup method, clearing zone	*Senna alata*	Leaf-hexane extract	12 mm	[222]
	*Colletotrichum gloeosporioides*	Percent inhibition at 1,000 ppm	*Senna spectabilis*	Leaf-dichloromethane Leaf-methanol	0.00 ± 0.00 1.85 ± 1.15	[223]
	*Colletotrichum gloeosporioides*	Percent inhibition at 1,000 ppm	*Senna spectabilis*	Flower-dichloromethane Flower-methanol	17.78 ± 1.73 2.59 ± 0.58	[223]
	*Colletotrichum gloeosporioides*	Percent inhibition at 1,000 ppm	*Senna spectabilis*	Stem-dichloromethane Stem-methanol	1.48 ± 1.15 15.93 ± 0.58	[223]
	*Curvularia lunata*	Inhibition zone (filter paper disc diffusion method)	*Senna occidentalis*	Whole plant ethanol extract	16-26 mm	[212]
	*Cryptococcus neoformans*	The cup plate agar diffusion method	*Senna alata*	Leaf hot water/leaf-methanol/leaf-acetone	3 mm/4 mm/3 mm	[205]
	*Cryptococcus neoformans*	The cup plate agar diffusion method	*Senna alata*	Root hot water/root-methanol/root-acetone	3 mm/4 mm/4 mm	[205]
	*Cryptococcus neoformans*	Minimum inhibitory concentration	*Senna alata*	Leaf-methanol/root-methanol	13 mg/mL/6 mg/mL	[205]
	*Cryptococcus neoformans*	Minimum microbicidal concentration	*Senna alata*	Leaf-methanol/root-methanol	13 mg/mL/6 mg/mL	[205]
	*Epidermophyton floccosum*	Agar diffusion and broth dilution method, minimum inhibitory concentration	*Senna alata*	Leaf-crude ethanol extract	3.75 mm	[208]
	*Epidermophyton floccosum*	Agar diffusion method	*Senna alata*	Ethanolic steam bark 5.00 mg/mL & 10 mg/mL	15.50 mm/20.05 mm	[206]
	*Epidermophyton floccosum*	Minimum inhibitory concentration	*Senna alata*	Steam bark-ethanol	5 mg/mL	[206]
	*Epidermophyton floccosum*	Minimum fungicidal concentration	*Senna alata*	Steam bark-ethanol	10 mg/mL	[206]
	*F. moniliforme*	Inhibition zone (filter paper disc diffusion method)	*Senna occidentalis*	Whole plant ethanol extract	12-36 mm	[212]
	*Fusarium oxysporum*	Percent inhibition at 1,000 ppm	*Senna spectabilis*	Leaf-dichloromethane Leaf-methanol	4.81 ± 1.115 7.04 ± 0.58	[223]
	*Fusarium oxysporum*	Percent inhibition at 1,000 ppm	*Senna spectabilis*	Flower-dichloromethane Flower-methanol	17.78 ± 1.73 19.26 ± 2.31	[223]
	*Fusarium oxysporum*	Percent inhibition at 1,000 ppm	*Senna spectabilis*	Stem-dichloromethane Stem-methanol	5.19 ± 0.58 44.44 ± 0.00	[223]
	*Helminthosporium oryzae*	Minimum inhibitory concentration	*Senna alata*	Aqueous flower extracts	15 mg/mL	[198]
	*Microsporum audouinii*	Minimum inhibitory concentration	*Senna alata*	Aqueous flower extracts	15 mg/mL	[198]
	*Microsporum canis*	Cup-plate method, mean zone of inhibition	*Senna alata*	Ethanolic leaf extract	14.4-30 mm	[207]
	*Microsporum canis*	Cup-plate method, mean zone of inhibition	*Senna alata*	Aqueous leaf extracts	17.20-32.0 mm	[207]
	*Microsporum canslaslomyces*	Agar diffusion method	*Senna alata*	Ethanolic steam bark 5.00 mg/mL & 10 mg/mL	12 mm/13.5 mm	[206]
	*Microsporum canslaslomyces*	Minimum inhibitory concentration	*Senna alata*	Steam bark-ethanol	5 mg/mL	[206]
	*Microsporum canslaslomyces*	Minimum fungicidal concentration	*Senna alata*	Steam bark-ethanol	5 mg/mL	[206]
	*Microsporum gypseum*	Agar diffusion and broth dilution method, minimum inhibitory concentration	*Senna alata*	Leaf-crude ethanol extract	10.42 mm	[208]
	*Microsporum gypseum*	Hyphal growth inhibition concentration (IC50)	*Senna tora*	Leaf-methanol	1.8 mg/mL	[209]
	*Microsporum gypseum*	Hyphal growth inhibition concentration (IC50)	*Senna alata*	Leaf-methanol	0.8 mg/mL	[209]
	*Microsporum gypseum*	Agar diffusion and broth dilution method, minimum inhibitory concentration	*Senna alata*	Leaf-crude ethanol extract	10.42 mm	[208]
	*Penicillium notatum*	Cup-plate method, mean zone of inhibition	*Senna alata*	Ethanolic leaf extract	19.4-30 mm	[207]
	*Penicillium notatum*	Cup-plate method, mean zone of inhibition	*Senna alata*	Aqueous leaf extracts	15.20-22.0 mm	[207]
	*Penicillium marneffei*	Hyphal growth inhibition concentration (IC50)	*Senna tora*	Leaf-methanol	1.8 mg/mL	[209]
	*Penicillium marneffei*	Hyphal growth inhibition concentration (IC50)	*Senna alata*	Leaf-methanol	6.6 mg/mL	[209]
	*Phytophthora parasitica*	Percent inhibition at 1,000 ppm	*Senna spectabilis*	Leaf-dichloromethane Leaf-methanol	−28.57 ± 0.00 −24.29 ± 2.65	[223]
	*Phytophthora parasitica*	Percent inhibition at 1,000 ppm	*Senna spectabilis*	Flower-dichloromethane Flower-methanol	−27.14 ± 1.99 −1.90 ± 2.31	[223]
	*Phytophthora parasitica*	Percent inhibition at 1,000 ppm	*Senna spectabilis*	Stem-dichloromethane stem-methanol	−17.62 ± 2.08 44.76 ± 1.15	[223]
	*Rhizoctonia solani*	Percent inhibition at 1,000 ppm	*Senna spectabilis*	Leaf-dichloromethane Leaf-methanol	0.00 ± 0.00 27.41 ± 0.58	[223]
	*Rhizoctonia solani*	Percent inhibition at 1,000 ppm	*Senna spectabilis*	Flower-dichloromethane Flower-methanol	47.04 ± 2.52 22.22 ± 4.58	[223]
	*Rhizoctonia solani*	Percent inhibition at 1,000 ppm	*Senna spectabilis*	Stem-dichloromethane Stem-methanol	37.78 ± 1.00 29.63 ± 0.00	[223]
	*Trichophyton mentagrophyte*	Cup-plate method, mean zone of inhibition	*Senna alata*	Aqueous leaf extracts	20.20-35.0 mm	[207]
	*Trichophyton mentagrophyte*	Agar diffusion and broth dilution method, minimum inhibitory concentration	*Senna alata*	Leaf-crude ethanol extract	19.64 mm	[208]
	*Trichophyton mentagrophyte*	Cup-plate method, mean zone of inhibition	*Senna alata*	Ethanolic leaf extract	16.4-30 mm	[207]
	*Trichophyton mentagrophytes*	Agar cup method, clearing zone	*Senna alata*	Leaf-hexane extract	14-18 mm	[222]
	*Trichophyton mentagrophytes*	Agar cup method, clearing zone	*Senna alata*	Leaf-chloroform extract	22-26 mm	[222]
	*Trichophyton mentagrophytes*	Agar cup method, clearing zone	*Senna alata*	Leaf-ethyl acetate extract	16-18 mm	[222]
	*Trichophyton mentagrophytes*	Agar diffusion method	*Senna alata*	Ethanolic steam bark 5.00 mg/mL & 10 mg/mL	17 mm/19 mm	[206]
	*Trichophyton mentagrophytes*	Minimum inhibitory concentration	*Senna alata*	Steam bark-ethanol	5 mg/mL	[206]
	*Trichophyton mentagrophytes*	Minimum fungicidal concentration	*Senna alata*	Steam bark-ethanol	5 mg/mL	[206]
	*Trichophyton rubrum*	Hyphal growth inhibition concentration (IC50)	*Senna tora*	Leaf-methanol	1.2 mg/mL	[209]
	*Trichophyton rubrum*	Hyphal growth inhibition concentration (IC50)	*Senna alata*	Leaf-methanol	0.5 mg/mL	[209]
	*Trichophyton rubrum*	Agar diffusion and broth dilution method, minimum inhibitory concentration	*Senna alata*	Leaf-crude ethanol extract	18.75 mm	[208]
	*Trichophyton verrucosum*	Agar diffusion method	*Senna alata*	Ethanolic steam bark 5.00 mg/mL & 10 mg/mL	15 mm/21 mm	[206]
	*Trichophyton verrucosum*	Minimum inhibitory concentration	*Senna alata*	Steam bark-ethanol	5 mg/mL	[206]
	*Trichophyton verrucosum*	Minimum fungicidal concentration	*Senna alata*	Steam bark-ethanol	5 mg/mL	[206]

Antiviral activity	*Herpes simplex*	Plaque-inhibition method, reduction factor was measured	*Senna occidentalis*	Whole plant-ethanolic extract	1 0 *μ*g/mL	[212]
	*HIV-1*	HIV-1 RT inhibitory assay, % inhibition ratio	*Senna alata*	Aerial part-ethanolic extract	35.86	[216]
	*HIV-1*	HIV-1 RT inhibitory assay, % inhibition ratio	*Senna alata*	Aerial part-water extracts	37	[216]
	*Coxsackie*	Plaque-inhibition method, reduction factor was measured	*Senna occidentalis*	Whole plant-ethanolic extract	1 *μ*g/mL	[212]
	*Measles*	Plaque-inhibition method, reduction factor was measured	*Senna occidentalis*	Whole plant-ethanolic extract	1 *μ*g/mL	[212]
	*Poliomyelitis*	Plaque-inhibition method, reduction factor was measured	*Senna occidentalis*	Whole plant-ethanolic extract	1 *μ*g/mL	[212]
	*Semliki forest*	Plaque-inhibition method, reduction factor was measured	*Senna occidentalis*	Whole plant-ethanolic extract	1 *μ*g/mL	[212]
	*Vesicular stomatitis*	Plaque-inhibition method, reduction factor was measured	*Senna occidentalis*	Whole plant-ethanolic extract	1 *μ*g/mL	[212]

Herbicidal activity	*Brassica chinensis*	Percent inhibition germination at 10,000 ppm	*Senna spectabilis*	Leaf-dichloromethane Leaf-methanol	12.66 ± 2.89 25.28 ± 7.77	[223]
	*Brassica chinensis*	Percent inhibition germination at 10,000 ppm	*Senna spectabilis*	Flower-dichloromethane Flower-methanol	71.38 ± 3.06 8.03 ± 0.58	[223]
	*Brassica chinensis*	Percent inhibition germination at 10,000 ppm	*Senna spectabilis*	Stem-dichloromethane Stem-methanol	6.90 ± 1.00 1.14 ± 1.53	[223]
	*Brassica chinensis*	Percent inhibition hypocotyl at 10,000 ppm	*Senna spectabilis*	Leaf-dichloromethane Leaf-methanol	68.09 ± 4.00 97.33 ± 1.31	[223]
	*Brassica chinensis*	Percent inhibition hypocotyl at 10,000 ppm	*Senna spectabilis*	Flower-dichloromethane Flower-methanol	99.67 ± 0.58 91.40 ± 1.31	[223]
	*Brassica chinensis*	Percent inhibition hypocotyl at 10,000 ppm	*Senna spectabilis*	Stem-dichloromethane Stem-methanol	−42.94 ± 5.18 34.75 ± 2.88	[223]
	*Brassica chinensis*	Percent inhibition radical 10,000 ppm	*Senna spectabilis*	Leaf-dichloromethane Leaf-methanol	84.48 ± 2.63 100.00 ± 0.00	[223]
	*Brassica chinensis*	Percent inhibition radical 10,000 ppm	*Senna spectabilis*	Flower-dichloromethane Flower-methanol	100.00 ± 0.00 100.00 ± 0.00	[223]
	*Brassica chinensis*	Percent inhibition radical 10,000 ppm	*Senna spectabilis*	Stem-dichloromethane Stem-methanol	−46.94 ± 7.82 99.94 ± 1.74	[223]
	*Chloris barbata*	Percent inhibition germination at 10,000 ppm	*Senna spectabilis*	Leaf-dichloromethane Leaf-methanol	72.71 ± 0.00 100.00 ± 0.00	[223]
	*Chloris barbata*	Percent inhibition germination at 10,000 ppm	*Senna spectabilis*	Flower-dichloromethane Flower-methanol	100.00 ± 0.00 95.50 ± 0.58	[223]
	*Chloris barbata*	Percent inhibition germination at 10,000 ppm	*Senna spectabilis*	Stem-dichloromethane Stem-methanol	4.50 ± 1.00 95.50 ± 0.58	[223]
	*Chloris barbata*	Percent inhibition shoot at 10,000 ppm	*Senna spectabilis*	Leaf-dichloromethane Leaf-methanol	85.31 ± 7.45 100.00 ± 0.00	[223]
	*Chloris barbata*	Percent inhibition shoot at 10,000 ppm	*Senna spectabilis*	Flower-dichloromethane Flower-methanol	100.00 ± 0.00 98.82 ± 2.68	[223]
	*Chloris barbata*	Percent inhibition shoot at 10,000 ppm	*Senna spectabilis*	Stem-dichloromethane Stem-methanol	38.82 ± 3.19 96.93 ± 4.59	[223]
	*Chloris barbata*	Percent inhibition root at 10,000 ppm	*Senna spectabilis*	Leaf-dichloromethane Leaf-methanol	88.18 ± 8.75 100.00 ± 0.00	[223]
	*Chloris barbata*	Percent inhibition root at 10,000 ppm	*Senna spectabilis*	Flower-dichloromethane Flower-methanol	100.00 ± 0.00 98.72 ± 1.79	[223]
	*Chloris barbata*	Percent inhibition root at 10,000 ppm	*Senna spectabilis*	Stem-dichloromethane Stem-methanol	25.56 ± 2.55 96.49 ± 3.53	[223]

↓: inhibition; HIV: human immunodeficiency virus; RT: reverse transcriptase.

**Table 4:** Summary of some clinical trials conducted on *Senna* spp.

Samples	Type of study/findings/results	Country	Ref
*Senna alata* (L.) Roxb.	Randomized controlled trial Trial registration: TCTR0180828004 Evaluating the use and safety of *S. alata* on bowel function recovery among women with gynecologic cancer 90 women candidates diagnosed with gynecologic cancer were randomly assigned to postoperative consumption (45 with *S. alata* tea and 45 with warm water) Usage of *S. alata* significantly reduced the time of first passage of flatus (mean difference: -8.5 h; 95% confidence interval: -3.7, -13.4 h) and time of first defecation (mean difference: -19.8 h; 95% confidence interval: -11.2, -28.5 h) compared with the controls The use of *S. alata* showed a positive impact during the postoperative care of gynecologic cancer patients	Thailand	[231]

*Senna*	Randomized controlled and crossover study Assessing the efficacy and safety of *Senna* versus polyethylene glycol in treating constipation in children The proportional formula was used to calculate the sample size and 28 patients were obtained Effectiveness of laxative therapy was evaluated by mean of a three-variable construct (a) Daily bowel movement (b) Faecal soiling (c) S clean abdominal X-ray The study was completed before the time because an interim analysis showed effective results of *Senna* (*p* = 0.026) The maximum daily dose of *Senna* and polyethylene glycol was recorded as 38.7 mg and 17 g *Senna* therapy showed promising results against constipation in children with anorectal malformation	Mexico	[232]

*Senna*	Comparative study Evaluating of *Senna* and other oral bowel medicines for treating constipation in pediatric oncology patients getting opioids The results of 5-year investigation demonstrated that 41.8% (*n* = 245) had blood cancer, 50.3% (*n* = 295) had solid cancer, and 7.9% (*n* = 46) had brain cancer out of 586 matched samples (age: 0-20 years, ave. age: 11.5 years) Initializing *Senna* therapy, over another oral bowel medication, reduced the subsequent risk of surrogate markers of problematic constipation. Adjusted effect of *Senna* on enema (hazard ratio, 0.31; 95% confidence interval, 0.11-0.91), abdominal radiographic imaging (hazard ratio, 0.74; 95% confidence interval, 0.55-0.98), and escalation of oral bowel medicine (hazard ratio, 0.78; 95% confidence interval, 0.59-1.03) were recorded	Philadelphia	[233]

*Senna*	Control single-blinded randomized study Assessing the efficacy and safety of gum chewing added to high dose *Senna* before colonoscopy promotes bowel cleaning 129 candidates participated and were further divided into two groups (a) *n* = 65 patients treated with *Senna* solution (150 mL) and sennoside tablet (80 mg) daily for 3 days before the colonoscopy (b) *n* = 64 patients were additionally advised to chew sugarless gum half an hour (three times) daily for 3 days The results demonstrated that gum chewing enhanced colonoscopy bowel preparation quality and is considered a physiologically sound, safe, and impassive part of the colonoscopy bowel preparation. The gum chewing group showed better cleaning compared to other groups	Turkey	[234]

*Senna*	Placebo-controlled, double-blinded, randomized study Evaluating the use of *Senna* with docusate for constipation after pelvic surgery 96 candidates completed a baseline seven-day bowel diary pre- and postsurgery. After pelvic surgery, candidates were divided into two groups: (a) *n* = 45 in the placebo group and (b) *n* = 48 in *Senna* (8.6 mg) with docusate (50 mg) group. The findings demonstrated that the use of *Senna* with docusate decreases the time to first bowel movement in those undergoing pelvic surgery than placebo (3.00 vs. 4.05 days; *p* = 0.001).	Philadelphia	[235]

*Senna*	Case study Case of a 31-year-old female patient who, after prolonged ingestion of *Senna* extract, developed severe weight loss, cyclic oedema, and dyspepsia, accompanied by an asymptomatic increase in markers of liver and muscle damage, dyslipidemia, electromyographic alterations, and mitochondrial myopathy in the muscle biopsy This clinical case is of particular significance, given that *Senna* is widely used for its pharmacological properties, with failure to consider its potentially toxic effects	Portugal	[236]

*Senna*	Single-blinded randomized study The effectiveness of *Senna* tables and sodium phosphate solution for bowel preparation before colonoscopy was examined for its efficiency A total of 134 candidates were treated with *Senna* tablets (180 mg) and sodium phosphate solution (95 mL) on the day before colonoscopy The results demonstrated that the mean cleanliness scores in the four segments of the colon (rectum, sigmoid segments, descending colon, and transverse colon) except the cecum were higher in the sodium phosphate group than in the *Senna* group (7.9 vs. 8.3, 8.0 vs. 8.5, 7.9 vs. 8.5, 7.9 vs. 8.2, and 7.2 vs. 6.9, respectively) The taste of *Senna* was more effective compared to sodium phosphate solutions	Thailand	[237]

*Senna tora* (L.) Roxb.	Experimental study Supplementation of *S. tora* fibre on the serum lipid profile of diabetic Korean patients was evaluated. *S. tora* fibre supplement of a combination of soluble fibre extracted from *S. tora* (2 g), alpha-tocopherol (200 mg), ascorbic acid (500 mg), and maltodextrin (300 mg) was prepared in a pack and given to a total of 15 candidates 2 packs per day up to 2 months The results demonstrated that *S. tora* fibre products were safe for consumption and additionally provided the necessary amount of dietary fibre for helping in the maintenance of lipid status in diabetic (type II) patients	Korea	[238]

*Senna*	Controlled randomized single-blinded study Evaluating efficiency and acceptability of high dose *Senna* tablets and its comparison with standard polyethylene glycol in adult patients 192 patients participated and were treated into two groups: (a) *n* = 91 in polyethylene glycol group and (b) *n* = 101 in *Senna* group The *Senna* tablet group showed acceptable results for colon cleansing and tolerance compared to the polyethylene glycol group (*p* < 0.001)	—	[239]

*Senna*	Controlled study Highly purified *Senna* extract was evaluated against cell proliferation, crypt length in the entire colon and gene expression (p53 and bcl-2). 171 patients (84 with sennoside-containing syrup and 87 without sennoside-containing syrup) were included 15 patients with *Senna* and 17 without *Senna* from 32 randomized patients were used for biopsies Proliferation activity in four areas of colon and gene expression (p53 and bcl-2) was evaluated by using 5-bromo-2′-deoxyuridine labelling, immunohistochemistry, and immunohistochemical The results demonstrated that crypts were shorter in the *Senna* group than without *Senna* group in the transverse and sigmoid colon. In the entire colon, the labelling index was higher in the *Senna* group than without the *Senna* group. In addition, bcl-2 expression was higher in both groups when crypts were shorter and proliferation was enhanced while no difference was recorded in p53 expression	Netherlands	[240]

*Senna* and MaZiRenWan (MZRW) (1st phase)	A double-blinded, double-dummy, randomized, and controlled trial Trial registration: NCT01695850 The protocol evaluated the effectiveness of MaZiRenWan (MZRW) with laxative *Senna* for functional constipation 291 candidates were recruited, and after a 2-week run-in period, the suitable candidates were randomly grouped into the three viz (a) Chinese medicine arm (MZRW and western medicine placebo) (b) Western medicine arm (*Senna* and Chinese medicine placebo) (c) Placebo arm (Chinese medicine placebo and western medicine placebo) The results of the eight-week treatment showed the increased responder rate for a complete spontaneous bowel movement (*CSBM*≧1/week) in the course of the treatment while the eight-week follow-up period showed changes of colonic transit, individual and global symptom assessments, and adverse effects	China	[241]

*Senna* and MaZiRenWan (MZRW) (2nd phase)	A double-blind, double-dummy, randomized, and controlled trial Trial registration: NCT01695850 Evaluating the efficacy and safety of Chinese herbal medicine MZRW and its comparison with the stimulant laxative *Senna* and placebo against functional constipation Primary and secondary outcomes demonstrated that the MZRW showed well-accepted effects in increasing complete spontaneous bowel movement per week compared to the *Senna* group (68.0% vs. 57.7%, with *p* = 0.14) during the treatment. After the eight-week-follow-up period, 47.4% of patients had a complete response to MZRW, 20.6% had a complete response to *Senna*, and 17.5% had a complete response to placebo (*p* < 0.005 for MZRW vs. placebo)	China	[242]

## Data Availability

The data supporting this review are from previously reported studies and datasets, which have been cited. The processed data are available from the corresponding authors upon request.
